# Evaluation of the National Health Insurance Program of Nepal: are political promises translated into actions?

**DOI:** 10.1186/s12961-022-00952-w

**Published:** 2023-01-20

**Authors:** Geha N. Khanal, Bishal Bharadwaj, Nijan Upadhyay, Tulasi Bhattarai, Minakshi Dahal, Resham B. Khatri

**Affiliations:** 1 Public Health Professional, Kathmandu, Nepal; 2 School of Earth and Environmental Science, The University of Queensland, Brisbane, Australia; 3 Ministry of Federal Affairs and General Administration, The Government of Nepal, Singadarbar, Kathmandu, Nepal; 4 Claim Reimbursement Department, Government of Nepal, Health Insurance Board, Teku, Kathmandu, Nepal; 5 Nepal Public Health Association (NEPHA), Lalitpur, Nepal; 6 School of Public Health, University of Queensland, Brisbane, Australia; 7 Health Social Science and Development Research Institute, Kathmandu, Nepal

**Keywords:** Health insurance, Health system, Reform, Nepal, Policy analysis, Political economy factors

## Abstract

**Background:**

Despite political promise to reduce out-of-pocket (OOP) expenditure on healthcare through the National Health Insurance Program (NHIP) of Nepal, its implementation is challenging with low enrolment and high drop-out rates. Program performance can often be linked with political economy considerations and interests of stakeholders. This study aimed to develop an in-depth understanding of organizational and systemic challenges in implementing NHIP.

**Methods:**

We conducted a structured narrative review of available literature on the NHIP in Nepal. We analysed data using a political economy analysis for health financing reform framework. The findings were explained under six broad categories: interest groups, bureaucracy, budgets, leadership, beneficiary and external actors. In addition, we triangulated and further presented the literature review findings using expert opinions (views expressed in public forums).

**Results:**

Nepal has formulated acts, rules, regulations, and policies to implement NHIP. Under this program, the Health Insurance Board (HIB) is the purchaser of health services, and health facilities under the Ministry of Health and Population (MoHP) are the providers. The NHIP has been rolled out in all 77 districts. Several challenges have hindered the performance of NHIP at the policy and implementation levels. Challenges under interest groups included inadequate or delayed reimbursement and drop-out of hospitals in implementing the programme. Bureaucracy-related challenges were hegemony of provider over the purchaser, and inadequate staff (delay in the approval of organogram of HIB). There was inadequate monitoring of premium collection, and claim reimbursement was higher than collected premium. Challenges under leadership included high political commitments but weak translation into action, consideration of health insurance as poor return on investment, and intention of leaders to privatize the NHIP. Beneficiaries experienced compromised quality of care or lack of services when needed, high drop-out rates and low interest in renewal of premiums. External actors provided technical assistance in policy design but limited support in implementation.

**Conclusions:**

Despite enabling a policy environment, the NHIP faced many challenges in implementation. There is an urgent need for institutional arrangements (e.g. digitalization of claims and reimbursement, endorsement of organogram of HIB and recruitment of staff), increased coverage of financial protection and service (increased benefit package and introduction of cost-sharing/co-payment model), legislative reforms (e.g. legal provision for cost-sharing mechanism, integration of fragmented schemes, tripartite agreement to reimburse claims and accreditation of health facilities to ensure quality healthcare), and leveraging technical support from the external actors. High levels of commitment and accountability among political leaders and bureaucrats are required to strengthen financial sustainability and implementation.

## Background

Nepal’s modern healthcare system has a seven-decades-long history. The end of the 104-year reign of the Rana Dynasty in the 1950s opened the gate to the modern healthcare system [1]. From 1960 to 1990, various health facilities (HFs) were established, and several vertical public health programmes and interventions were introduced. In the early 1990s, with the end of the Cold War and with economic liberalization, Nepal shifted its policy towards private investment in healthcare [2]. In 1991, the National Health Policy was endorsed, which led to improving access to basic healthcare services (BHS) through the establishment of health posts (HPs), primary healthcare centres (PHCCs), and district hospitals [3]. Nepal has adopted two types of health delivery systems: publicly funded and private with paid-for services. The federal system of 2017 established three tiers of government (federal, provincial and local) [4]. Currently, the public health services are delivered through a wider network of 7221 public HFs (125 hospitals, 205 PHCCs, 395 Ayurvedic hospitals, 3870 HPs and 2626 community health centres), which fall under the jurisdiction of any of these three tiers of government [5]. In addition, private health institutions (hospitals, nursing homes, clinics and private medical shops) have provided private healthcare services since the late 1990s.

Nepal has adopted a mixed healthcare financing system in line with the healthcare delivery system. There are government-funded health programmes for BHS and insurance-based tertiary services, and private health services through out-of-pocket (OOP) expenditure. The BHS is provided free of cost through public facilities and paid-for services beyond the BHS package in those facilities. The government pools the tax-based healthcare fund for conditional, fiscal equalization, complementary or special grants [6, 7]. Sources of health financing at the federal level include domestic revenues, foreign aid or internal borrowing. Health financing sources at the provincial and local levels are arranged through fiscal transformation, revenue sharing, internal sources, and internal borrowing or federal loans [7]. External development partners (EDPs) either support government healthcare programmes directly through earmarked funding or assist in the implementation of health programmes through nongovernmental organizations [8, 9].

In Nepal, public healthcare facilities are often criticized for being of compromised quality, often lacking medical supplies, equipment, competent and motivated health workforce, and services [10]. Consequently, people opt for private providers (even BHS) regardless of their economic status [11]. The OOP constitutes a major source of financing (57.7%) of current health expenditure (CHE), while the governmental share of CHE is only 22.6% [12]. Total external funding for health (through direct foreign transfers or foreign transfers distributed for health through the government) constitutes about 13.5% of CHE [12]. More than three quarters of total OOP spending is used for pharmaceuticals and medical supplies. Healthcare cost at hospitals constitute 20.8% of total OOP, and 80% of such spending occurs in private hospitals [12].

About half a million of the Nepalese population are pushed below the poverty line each year just because of expensive healthcare [12]. Nepal has introduced different social protection schemes to reduce such catastrophic expenditures. These schemes aim to improve the health status of people, and include voluntary private insurance, Social Security Fund (SSF), Employee Provident Fund (EPF) and enterprise private insurance, free healthcare, conditional cash transfer programmes, impoverished citizens’ programme and National Health Insurance Program (NHIP) [13–16].

NHIP is the most recent healthcare financing programme designed after the political mandates of the popular movement in 2006. With the thrust of the pro-socialist constitution of 2015, all political parties promised to provide BHS free of charge for all citizens and services beyond BHS through NHIP [17]. People with high OOP risk falling into the poverty trap, as the existing healthcare system cannot fully protect the marginalized groups. The Health Insurance Policy was endorsed in 2014 [18], and the Social Health Security Development Committee (SHSDC) was established to implement NHIP.

The NHIP is a social health protection programme implemented by the Government of Nepal to provide quality healthcare to its citizens without financial hardship. The programme is designed to help prevent people from becoming impoverished due to healthcare expenditures incurred because of accidents or illness [19]. NHIP also promotes high-quality healthcare for all, by addressing barriers to service utilization and ensuring equity and access for all. The NHIP aims to achieve universal health coverage (UHC) by reducing impoverishment and catastrophic expenditure in seeking healthcare [9]. NHIP was piloted through three districts (Kailali, Baglung and Ilam) in 2016 [20].

**Fig. 1:**
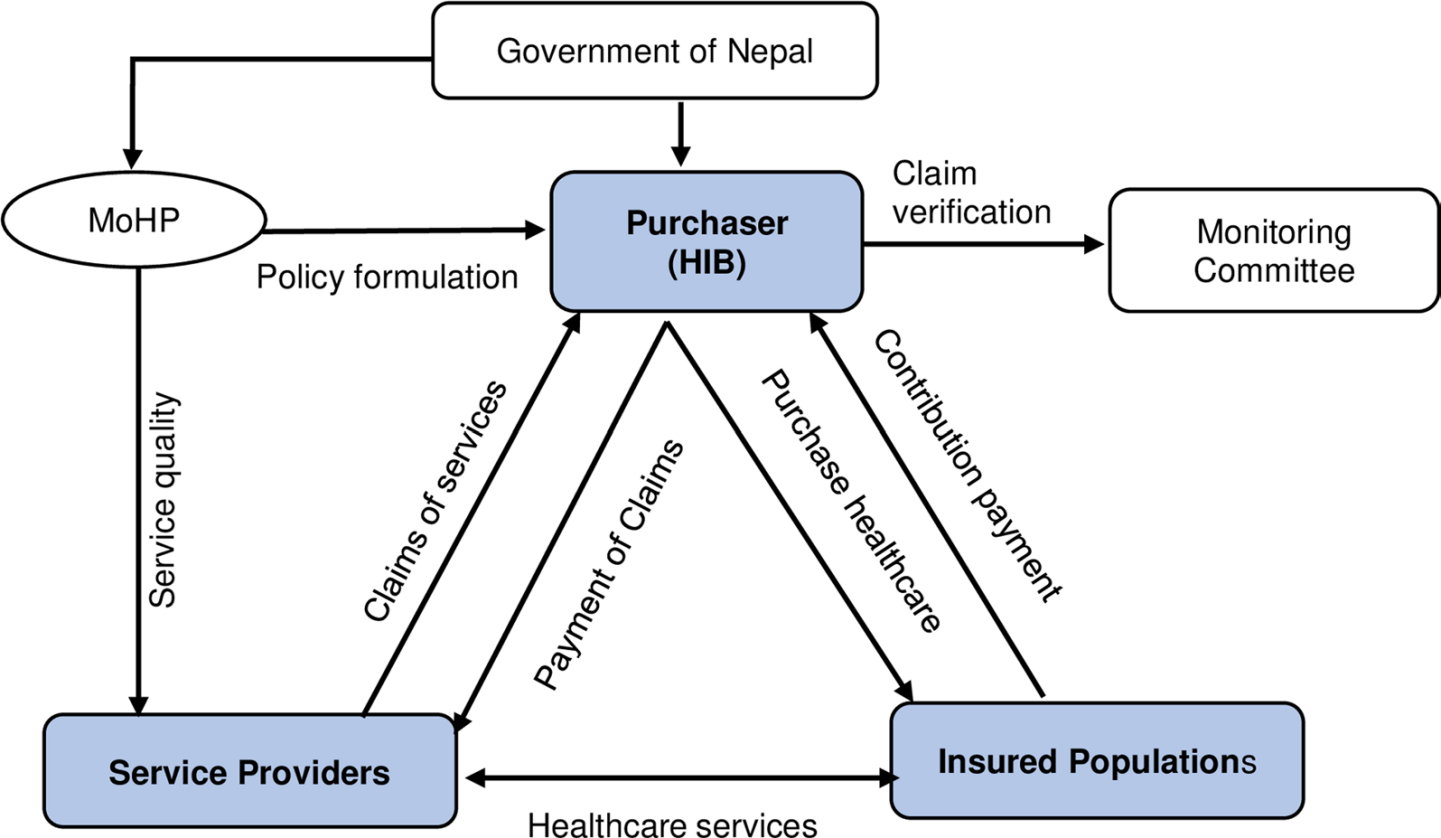
Purchaser–provider split in NHIP in Nepal. Source: Prepared by the authors (GNK and RBK), adapted from the Health Insurance Training Manual [23]

After learning from the pilot programme, a separate Health Insurance Act (HIA) was enacted to govern the programme. Thus, in 2017, the parliament endorsed the HIA, which replaced the SHSDC with the Health Insurance Board (HIB) [6]. The roles of HIB include raising revenue, pooling funds for financial risk protection, health and financial risk (funds received from contribution premiums, tax funding and other external funding), and organizing and purchasing from public and private providers [21]. Additionally, the HIA and Health Insurance Regulation (HIR) provide legal provisions and strategies for implementing NHIP [22]. HFs under the Ministry of Health and Population (MoHP) are the service provider, while HIB is the agency for purchasing healthcare services (Fig. 1).

Several legal and policy provisions (e.g. constitution, national laws, regulations and policies) guide the implementation of NHIP. Despite a favourable policy environment, performance indicators such as coverage (enrolment) and retention (renewal rate) are unsatisfactory [24]. The government had aimed to enrol 100% of the population in NHIP by 2022; however, only 21.4% enrolment had been achieved by mid-June 2022 [25, 26]. Likewise, the annual drop-out rate is about 25% [24], which is a serious concern.

Several political economy factors can influence the design and implementation of NHIP. For example, Nepal has a mixed health system with profit-motived and poorly regulated private provider dominance in tertiary health services [2, 27]. The instability of political leadership, frequent transfer of bureaucrats, and conflict of interest between the private sector and politicians are other macro-level systemic factors influencing health policy-making and health system functioning [28].

Furthermore, beneficiaries (e.g. health service users) may bypass local HFs in seeking high-quality care in facilities of urban areas, resulting in overcrowding of referral facilities [29, 30]. EDPs also play a role in Nepal’s health systems, including funding and technical support in policy and programme implementation [31]. In this context, an in-depth understanding of policy processes, power interactions, political forces, and resource allocation and distribution is essential to understand such unsatisfactory achievements [32]. The political economy analysis for health financing reform framework can guide the analysis of policy and programme implementation issues, including power distribution over access and control of resources, concerns with distribution of wealth and resources through power interactions among different stakeholders, and analyses of the interlinkage of political determinants [32]. This study synthesizes the factors of the political economy of health influencing policy and implementation of NHIP and suggests potential strategies for improving the uptake of health insurance services. The findings of this study will help to develop a detailed understanding and make necessary recommendations for Nepal’s health insurance programme.

## Methods

### Study design

We carried out a structured narrative review of available literature regarding the NHIP of Nepal. We extracted data from selected documents and determined their relevance using different components of the framework. Following the literature review, we validated and triangulated findings by exploring the views and opinions of experts in Nepal’s health system in general and health financing in particular [33]. We used publicly available information provided by the experts (in seminars, conferences and meetings), policy-makers and stakeholders of Nepal’s health system (see names in the acknowledgements section).

### Framework for data synthesis and analysis

We adapted Sparkes and colleagues’ framework on the political economy analysis for health financing reform to guide the analysis of the content [34]. This framework comprises the politics of six major stakeholders (interest groups, bureaucracy, budgets, political leadership, beneficiaries, and external actors or development partners) [34]. Broadly, the dimension of interest groups covers the roles and positions of governmental stakeholders (e.g. medical professionals, health service providers, health facilities and healthcare services). Budgetary politics refers to allocation of the budget and its expenditure mechanisms, absorption/distribution, expenditure and transparency mechanisms [34, 35]. Bureaucratic politics focuses on governmental agencies' financial, administrative or personnel authority and their interrelationships. Leadership determines strategic vision, technical knowledge, political skills and ethical orientation influenced by the perception of political benefits [34, 35]. Beneficiary politics considers beneficiaries' behaviours, preferences, and political activity within the system. Finally, external actor politics refers to funds and policy ideas generated by external actors in determining policy formulation [34, 35]. We framed our analysis considering these six dimensions of Nepal’s health insurance programme which could potentially be helpful in developing appropriate strategies for implementation.

### Selection strategy

We used search terms under two themes: health insurance (search terms were “health insurance” OR “social health protection” OR “national health insurance” OR “social health insurance”) AND location (Nepal). Using those search terms, we searched peer-reviewed studies in PubMed, Embase and Google Scholar. We also looked at references of selected papers to locate further studies that were not captured from database searches. Furthermore, we looked at the webpages of different ministries and entities under the Government of Nepal to access national acts and regulations, national policies, directives, circulars/notices, guidelines, conference presentation/panel discussion videos, assessment/survey reports, budget speech and red book, newspaper/magazine reports and political manifestos of major political parties. We consulted with subject experts using our personal network to identify additional relevant documents.

### Inclusion and exclusion criteria

We included the relevant literature for review in the context of our purpose, rather than the context of the primary studies. We focused on the content of these studies to answer our review question. We included grey literature published before July 2022, written in English or Nepali. We excluded those studies with irrelevant content or beyond the dimensions outlined in the Sparkes and colleagues’ framework.

**Fig. 2:**
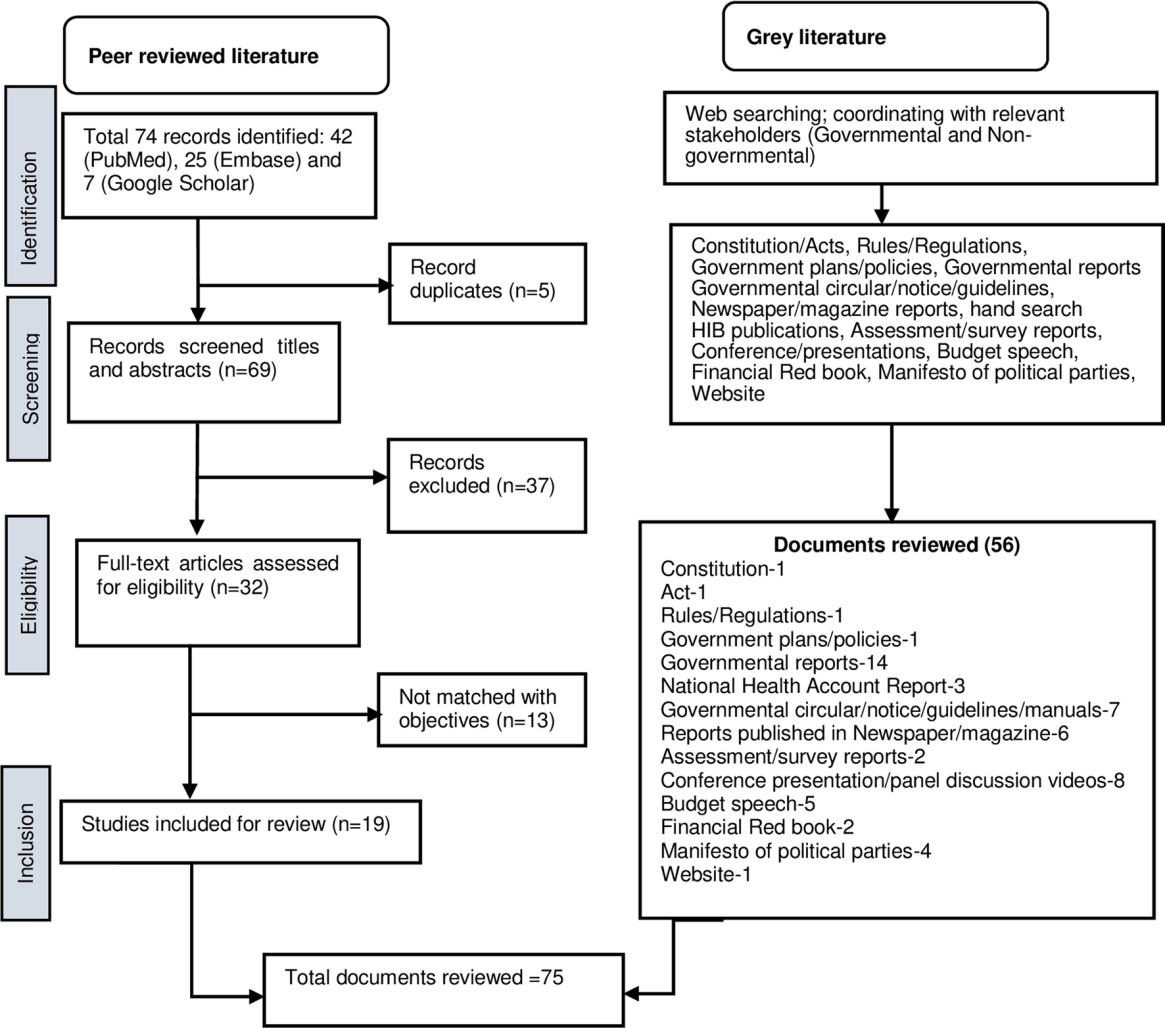
Flowchart of studies included in the study

Figure 2 presents the total documents that we reviewed for this study. Of the total 75 documents, 19 were peer-reviewed studies related to health insurance programmes of Nepal, while the remaining 56 documents (e.g. Constitution, acts, rules/regulations, guidelines, circulars, notices, manuals, budget speeches, red book, government reports, assessment reports, conference presentation and manifestos) were grey literature that was associated with the NHIP of Nepal.

### Data extraction and analysis

We critically reviewed the available literature to identify the stakeholders and their roles in implementing NHIP. We extracted relevant content (in line with the modified framework adapted for our analysis) and issues related to NHIP from the selected studies/reports. The extracted data were discussed among authors for further analysis and interpretation. We used a deductive content analysis approach (extracted data and fit extracts into a pre-identified framework). We conceptualized and interpreted the text according to each stakeholder/group of the framework. The first author conducted coding and analysis and discussed if inconsistencies were found. The findings of the literature review were shared with a few experts working with NHIP, and the content was synthesized according to the framework.

## Results

After 6 years of implementation, the NHIP has been expanded into 77 districts and 745 local governments [25]. By the end of July 2022, more than 5.66 million people (21.35% of the total population) from 1.74 million households (32.14% of total households) had been enrolled in the NHIP [36]. Table 1 presents a summary of dimension-specific stakeholders and their roles in the policy and implementation of NHIP, current implementation status and challenges.

**Table 1:** Category-specific stakeholders and their expected roles and implementation status, issues and challenges of NHIP

Categories	Stakeholders/institutions	Expected roles in the programme	Implementation status, issues and challenges
Interest groups	Medical professionals Healthcare service providers Public and private hospitals Other insurance providers General public	Service contract with eligible hospitals for expansion of NHIP [37] NHIP service costing [38]	The interest of private hospitals: After accreditation with NHIP, they have increased revenue Demanded revision of the reimbursement rate set in 2017 Interest of public: Tendency to enrol if they have a chronic illness Tendency to seek healthcare just because they have an insurance policy Insurance company: No role of private insurance companies in NHIP yet Have lobbied policy-makers to privatize the NHIP [39] The thrust of social health security and risk-sharing among the poor is not possible with such privatization Politicians: Inadequate commitment to health sector structural reform Had planned (later withdrew after public pressure) to hand over the institutional management of NHIP to the private sector [40] Limited understanding of fundamentals of social health security and its prerequisites Healthcare providers: Reluctant to strengthen public healthcare system as it might affect the revenue generated through private practice
Bureaucracy	Federal, provincial and local governments Ministries (MoHP, MoF, Ministry of Federal Affairs and General Administration) HIB Legislative bodies	Recruitment of enrolment assistants (EAs) by local governments [22] Strengthening delivery of quality healthcare [41] Services by provincial and local governments [42] Enrolments and service expansion, ensure service quality [42] Identification of poor households [25] Overall execution and management of NHIP [22] Formulation and amendment of standard operating procedure (SOP), guidelines, acts and regulations [6, 17, 22] Autonomous role of HIB instead of being controlled by MoHP [43]	Legal frameworks: Endorsement of HIA in 2017 and HIR in 2019 [6, 22] Legal frameworks and procedural documents envisioned by HIA and HIR are not formulated yet [25, 44] Private practitioners within the premises of public hospitals: No restriction to establishing pharmacy, lab or hospitals within/adjoining public hospitals Degrading the quality of public health facilities (sometimes with intention) to secure the market of private practice Institutional development: HIB lacks a separate organizational and human resource structure as envisioned in HIA [6] Staff under HIB are deputized from MoHP [19] Programme implementation and expansion: Expanded to all 77 districts and 745 (out of 753) local governments (except urban municipalities of Kathmandu and Lalitpur district) [25] Poor household identification: Accomplished in 26 districts [45]; however, it was heavily criticized HIB planned to identify poor households in 12 additional districts in 2019/2020 but have not accomplished [47] Claim and reimbursement system: Delay in reimbursement of claims to the hospitals [42] Manual and random process of claim review Limited human resources (about 20) [46] Need to review more than 25,000 claims each day [47] Excessive workload due to high volume of claims, limited workforce and manual process High possibility of fraudulent claims from hospitals [48]
Budgets	Governmental leadership MoF MoHP HIB General public	Resource allocation for NHIP [6] Integrating fragmented health financing schemes [9] The flexibility of ministerial leadership [49] MoHP for delivering quality, equitable and accessible healthcare [50] Organizational capacity of HIB for budgetary allocation and absorption [25] Assurance of financial sustainability [25, 51] Timely payment of annual premium [22]	Service costing: Service costing was revised (partially) in 2022 [52, 53] Service contract with HIB: 440 service providers in 77 districts [54], and the majority (82.5%) are public health facilities Some tertiary hospitals denied accreditation in NHIP Stagnation of OOP expenditure: High OOP expenditure (57.7%) [12] which has remained stagnant between 55% and 58% since 2009 [55, 56] Fragmented health financing schemes: Fragmentation of social health protection schemes like free healthcare, health insurance, conditional cash transfer and cost subsidization schemes under MoHP [9, 57] Gaps in budget allocation versus expenditure: More than one third of the allocated budget remains unspent [58–60] Unspent resources are pooled back by the central treasury [61] The practice of budget virement in other headings [49] Collection and mobilization of funds: The premium collected is deposited in the Health Insurance Fund (HIF) [6] Procedural documents are still lacking to operationalize HIF [62] The nonoperational amount in HIF doubled from NPR 1.4 billion to 2.8 billion between fiscal years 2019/2020 and 2020/2021 [63] HIB has to depend on the federal budget while its fund (HIF) has remained nonoperational [62] No clear guidelines for provincial and local governments to contribute in HIF [62] Doubtful financial sustainability: Widening gap between premium collection and reimbursement over time [63] Reimbursement exceeding the premium indicates possible threats to financial sustainability [51, 62, 64]
Leadership	Ruling party Opposition parties	Leverage political commitments [49] Facilitate a supportive political environment with strategies, institutions and structures [49]	Strong political commitment: Both ruling and opposition parties have a commitment to enrol citizens in NHIP [65, 66] Poor actions towards fulfilling the commitments of health system strengthening: Communist Party of Nepal (CPN) committed to establish a 25-bed hospital at a minimum in each 753 local government level [65]; however, only nine additional hospitals and five PHCCs were established during their tenure of 3.5 years [67], was not increased in sanctioned positions for medical officers in the last 4 years [47]
Beneficiaries	General public and targeted population (female community health workers, leprosy, multidrug-resistant tuberculosis, people living with HIV, null disability, senior citizens or ultra-poor families)	Regular enrolment and renewal [22] Rational use of NHI services [22]	Poor enrolment and higher drop-out: Economically better-off households are more likely to enrol in NHIP [68] 5.66 million (21.35% population) have enrolled in NHIP [36] Drop-out rate is about 25% [24] An early study in 46 districts showed that the drop-out rate was more than 38% [69] A study in Pokhara showed that more than a quarter (28.2%) of households dropped out from NHIP [70] More than one third (33.6%) of active insured (39.5 million) belong to the targeted population, whose premium was paid by the government [25] Major reasons behind drop-out were health service underutilization, poor healthcare services and inadequate benefit packages [25, 71] A high proportion of drop-out and subsidy enrolment is a key challenge for the sustainability of NHIP [25, 51] Low value for money: Beneficiaries receiving suboptimal service quality [19, 42] Service payment on the activity-based model rather than quality [4] Insured are willing to renew their membership if service quality is improved [72–77] or they are made aware of the renewal process [70]
External actors	External development partners: (GIZ, KOICA-Nepal Health Insurance Support Project, Save the Children, ILO, etc.)	Leverage technical expertise and financial resources	Leveraging donors and learning replication: KOICA-Nepal: Capacity-building of officials through international exposure visit [78]; technical assistance in development of e-learning package; preparation of mid-term and long-term strategy of HIB [24] Save the Children: Expansion of NHIP [24] GIZ: Development of SOP, guidelines, insurance management information system (IMIS), customize IMIS, training and orientation related to health insurance and open IMIS, international exposure visit to HIB/MoHP delegate, human resource support in information technology and claim system [24] ILO: Actuarial analysis Funding sustainability: Funding through KOICA-Nepal and Save the Children have ended from 2021 No other new partners have supported NHIP Technical assistance priority of EDPs: Foreign, Commonwealth & Development Office/UK Aid fund in strengthening basic healthcare services (tax-funded) GIZ and KOICA in social health insurance programme (German model or Korean model)

### Interest groups

HIB must accredit HFs for NHIP services [22]. Hospitals have demonstrated different levels of interest in whether to seek accreditation by the NHIP. Accreditation of private hospitals could increase patient flow and ultimately revenue. Due to the information asymmetry of patients (regarding the procedure and cost of treatment), private hospitals tend to make longer hospital stays, prescribe unnecessary tests, and make fraudulent claims and sometimes even double claims (claims from both HIB and patient party) to maximize their profit [42]. If all listed medicines were available with NHIP service providers, the insuree would rarely visit private pharmacies [79]. Since the HIA restricts accreditation of private pharmacies for providing NHIP, they perceived a loss in their business. Recently, private pharmacies have requested that HIB lift such legal constraints. In contrast, some public hospitals have hesitated to obtain accreditation for NHIP services. Possible reasons for this reluctance were to avoid unwanted referral cases, low reimbursement rate offered by HIB (compared with the hospital’s own rate) and disallowance made by HIB against their claims [48].

HIB has adopted the average costing method to finalize the reimbursement rate. Initially, HIB discussed finalizing this rate with public and private hospitals. Although HIB partially revised the reimbursement rate in 2021, the detailed costing has not yet been started [52, 53].

The service providers opted to claim from NHIP if the reimbursement rate was higher than other vertical programmes. For instance, HIB reimburses NPR 18,000 (nearly 137 USD) for caesarean section delivery [52], while the Safe Delivery Incentive Program (SDIP) reimburses NPR 7000 (53 USD) for the same services [80]. Consequently, some facilities terminated their contract with SDIP (stopped SDIP services) and opted for the NHIP. Similarly, some hospitals providing specific services (e.g. cataract surgery and dialysis) are attracted to NHIP due to the high reimbursement rate offered by HIB compared to their rate.

HIB has authority to review and approve reimbursement of claims, but the hospitals have strong reservations against disallowances. For example, a report revealed that claims of NPR 260 million from B.P. Koirala Institute of Health Sciences (BPKIHS) and NPR 120 million from Tribhuvan University Teaching Hospital were not reimbursed by HIB on time [81]. As a result, many large hospitals (both public and private) have declared that they are pulling out of the NHIP, and accused HIB of failing to reimburse their claims on time [82]. Such temporary cessation of NHIP services has created mistrust among the insured, who doubt the programme’s sustainability.

### Bureaucracy

HIB has extended federal, provincial and local networks. The federal-level organogram has different sections, the claim review and evaluation committee and dispute resolution committee [6]. There are provincial and district offices and coordination committees.

**Fig. 3:**
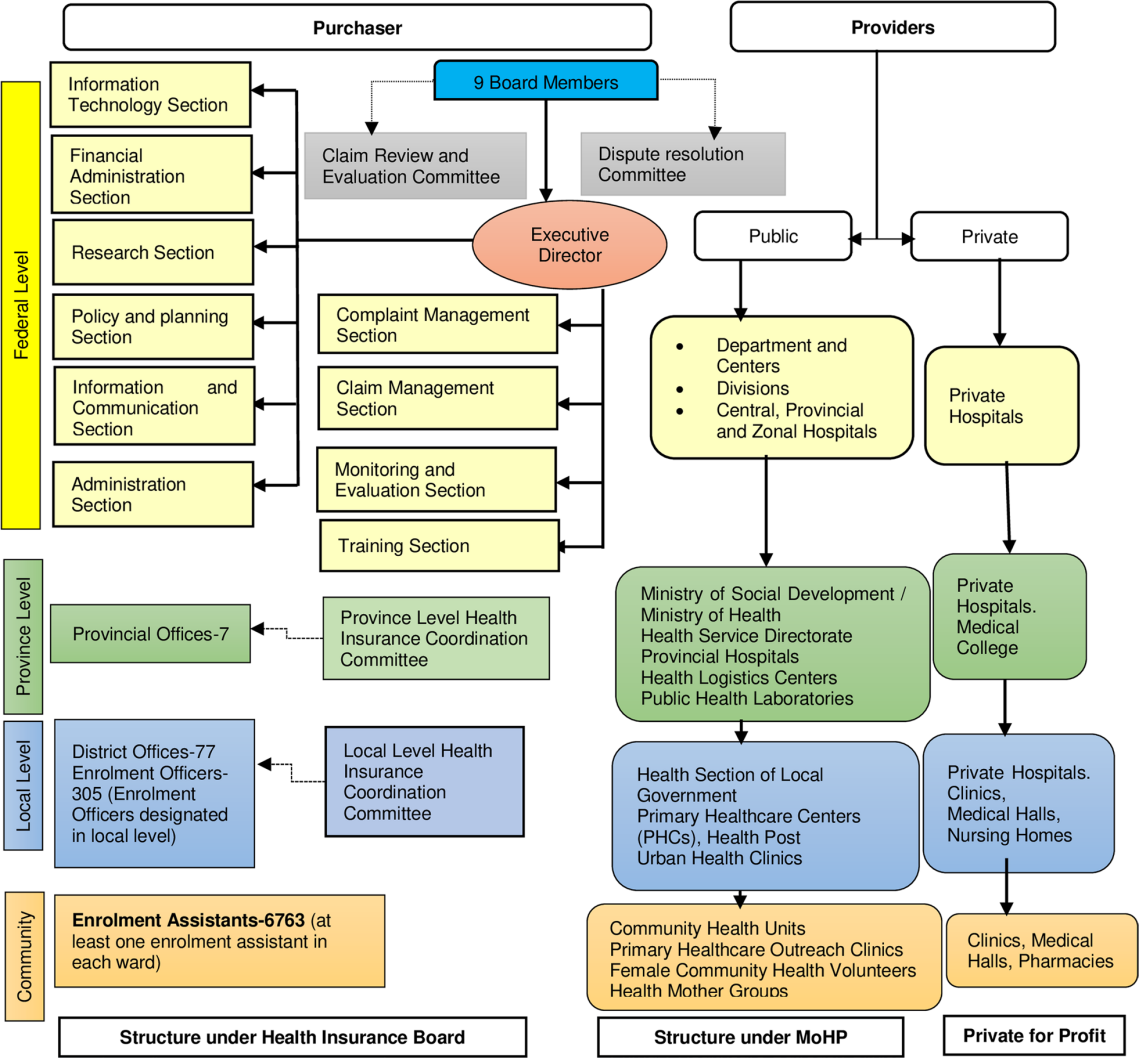
Schematic diagram of organogram of HIB (proposed) and health system of Nepal. Source: The authors (GNK and RBK), prepared using information from [22, 83, 84]

Local governments recruit enrolment assistants (EA) from each ward (catchment area) [22]. These EAs receive 3-day training and conduct home visits to explain the NHIP, enrol households and collect the premium [23]. The EAs are not salary-based staff but are incentivized with a commission (10% of the premium amount) [19]. Local stakeholders are engaged in enrolment, entry, premium collection and community awareness activities, while provincial and federal structures are involved in monitoring and quality assurance. Figure 3 shows the structure of HIB in line with the federalized health system.

According to the HIA, HIB is an autonomous entity responsible for purchasing and managing NHIP services. But in reality, HIB is indirectly placed under the jurisdiction of the MoHP [19, 50]. For instance, MoHP nominates the board members of HIB, examines the balance sheet and approves the organization and management (O&M) structure. Earlier, for no reason, MoHP delayed in addressing the HIB proposal to approve the human resource structure and financial procedures. Furthermore, MoHP has authority to nominate (and terminate) the majority of HIB board members [6]. In addition, HIB requires coordination with other ministries exclusively through the MoHP channel [6]. Recently, MoHP opposed increasing the number of hospitals or health workforce (even after 6 years of NHIP implementation) [5, 50]. This evidence suggests that MoHP perceives HIB as one of its entities regardless of the autonomous status of HIB (at least on paper). This has ultimately affected the quality of services under NHIP.

As of March 2022, a total of 440 HFs had been recognized as accredited service providers for NHIP: 346 are first service points (FSPs), and the remaining 94 are referral hospitals [54]. The FSPs are the HFs that the insuree (must) select during the enrolment process. Only public facilities (hospitals or PHCCs) can act as FSPs. The insuree needs to visit the FSPs for general check-ups. These FSPs (only) can refer the patients to referral hospitals (both public and private) if required. The insuree can visit referral hospitals only in emergency or referral cases; however, they need to obtain prescribed referral slips from FSPs for any kind of referral services [6].

Inadequate human resource is another challenge for the implementation of NHIP. Although the programme has expanded throughout the country, except executive director of HIB, all positions are managed through temporary deployments where only 372 positions are filled [71]. Earlier, HIB called a public notice to fill the temporary positions; however, MoHP denied providing approval without further notice [42]. Thus, HIB has been unable to recruit its staff even after 6 years of initiation NHIP implementation.

The bureaucratic function within HIB depends on the three levels of government. The local governments have roles in the recruitment of EAs. The MoHP (via HIB) has their role in forwarding organizational structure to parliament for endorsement, HIB formulates standard operating procedure (SOP), guidelines and standards, while at the federal level, the legislative parliament (via MoHP) can amend the HIA and HIR, respectively. However, integrating the functions of each tier of government is still challenging.

### Budgets

**Fig. 4:**
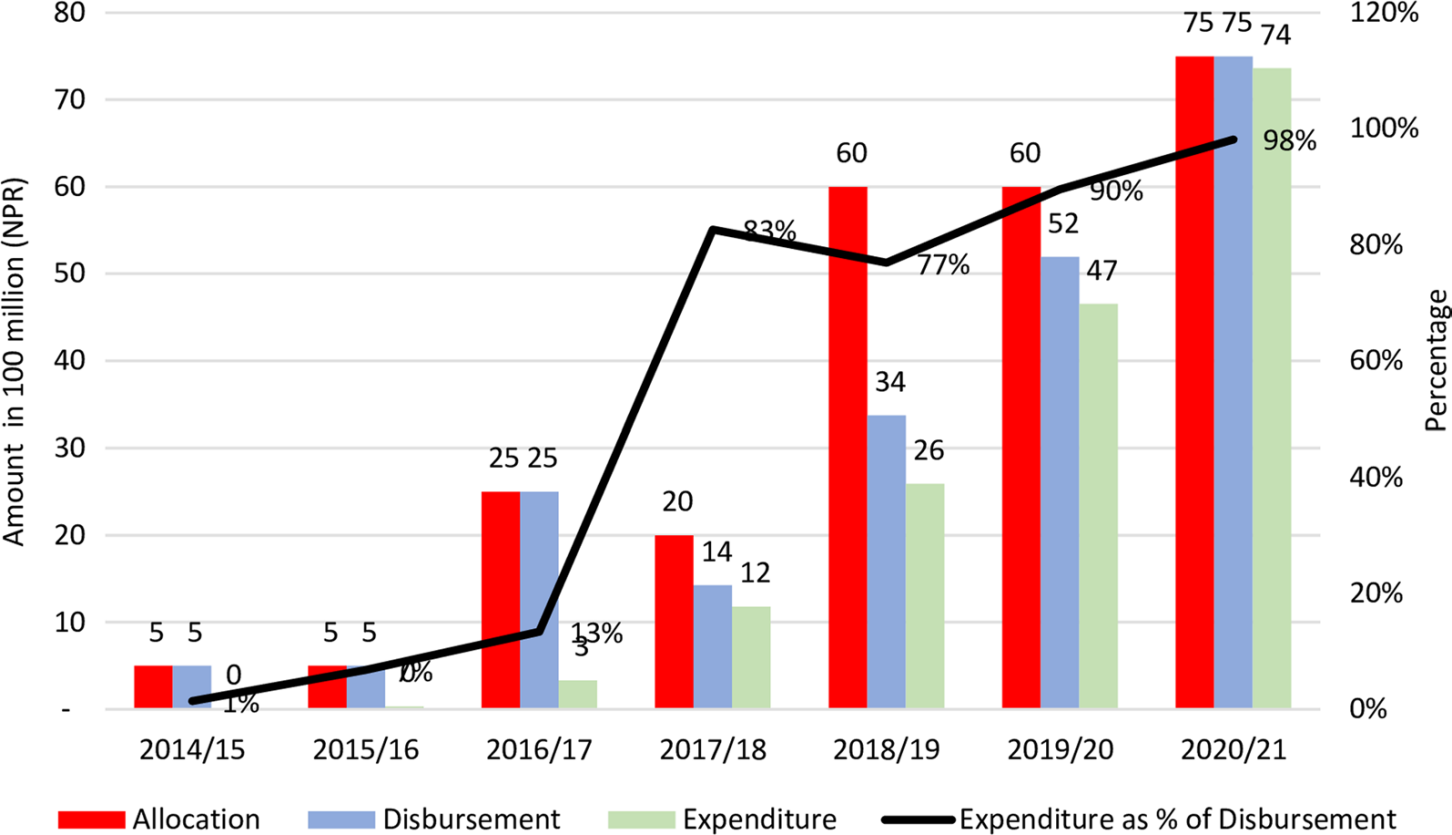
Allocation, disbursement and expenditure of NHIP budget in Nepal (2014/2015 to 2020/2021). Source: Prepared by the authors (GNK and RBK) using information from different documents [24, 58, 59, 86]

Figure 4 presents the allocation, disbursement, expenditure, and expenditure as a percentage of the disbursed budget for NHIP between fiscal years 2014/2015 and 2020/2021. In recent years, the disbursed amount has been lower than the allocation, while expenditure as a percentage of disbursement has increased.

The federal government is a major source of financing for NHIP. Provincial and local governments can also make financial contributions to NHIP. However, the policy provisions for local and provincial governments are still inadequate [63]. Some local and provincial governments have allocated resources to NHIP; however, their financial contribution seems invisible due to poor reporting systems [19].

Poor budget absorption capacity is one of the major criticisms faced by NHIP. The allocated budget for NHIP increased from 2 billion to 7.5 billion between 2017/2018 and 2020/2021, while the disbursement was lower than the allocation. The expenditure as a percentage of total disbursement was below 90% until fiscal year 2019/2020 [26, 85], and increased to 98.2% in fiscal year 2020/2021 [58, 59]. The disbursement amount was only 56.2% of the allocated amount in fiscal year 2018/2019. HIA allowed to provide for subsides in premium for the ultra-poor; however, the authentic data and procedural documentation to enrol poor and marginalized populations are still lacking. Hence, HIB is unable to pay the allocated premium amount for the poor. The lack of procedural legal documents also affects budget expenditure. After the mid-fiscal year, allocated budgets are reclassed to other headings and reallocated in the heading of political interest (of leaders) through the budget virement process [26].

Fund pooling (including premium collection) occurs through different mechanisms. The federal government pays the premium for targeted populations, while provincial and local governments and some nongovernmental organizations also pay the premium for poor and marginalized people [19]. The providers claim the expenses through the insurance management information system (IMIS) and are later reimbursed after necessary verification from HIB.

**Fig. 5:**
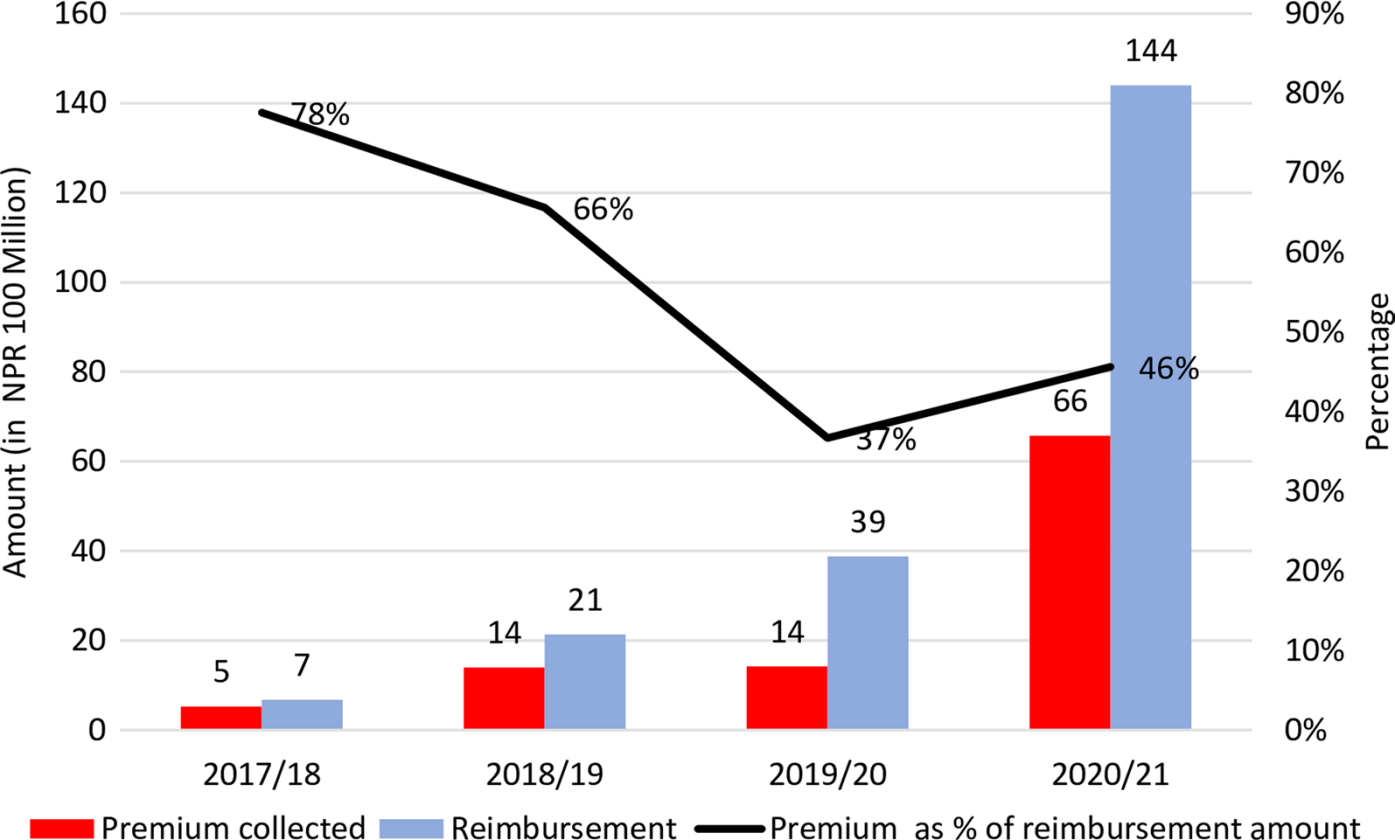
Reimbursement versus premium of health insurance programme in Nepal. Source: Prepared using information from [62–64, 87]

Figure 5 presents the analysis of premiums and reimbursement under NHIP between 2017/2018 and 2020/2021. During this period, the proportion of premium relative to the total reimbursement gradually decreased from 78% to 46%. These figures indicate that the amount reimbursed has outweighed the collected premium. NHIP was designed with the aim that the premium would cover healthcare expenditures. However, the trend of reimbursement versus premium indicates that NHIP has to rely on external resources to reimburse the claims. Such outpacing will increase the liability to the federal government, which would ultimately affect the programme's sustainability. This challenge would be exacerbated with the increase in service coverage.

### Leadership

Over the past three decades, Nepal has observed several political reforms and changes in the international paradigm of health service provision. Based on the thrust of primary healthcare, the National Health Policy (1991) provided policy provisions to establish HPs in each village. The MoHP later implemented free healthcare in 2006 and the NHIP in 2016 [17]. These policy and programme milestones demonstrated the political vision translated into actions to improve access to health services [88].

**Fig. 6:**
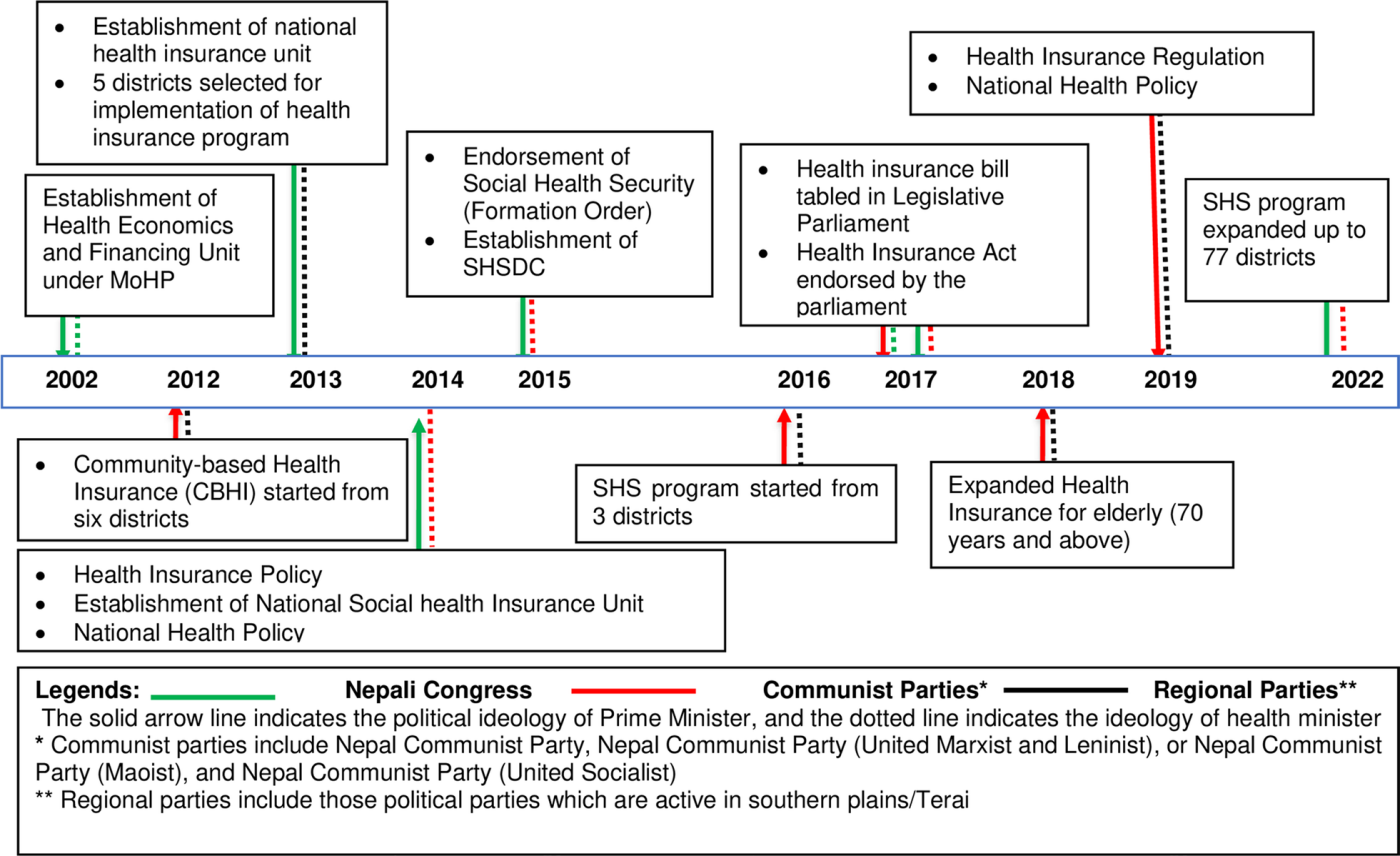
Political leadership in the development of NHIP in Nepal. Source: The authors (GNK and RBK), prepared based on available literature [22, 25, 89]

Figure 6 shows the policy trajectory in the past two decades in line with political ideology and political leadership in the development of the health insurance programme of Nepal [22, 25, 89]. Since 2000, Nepal has gone through several coalition governments with differing political ideologies on the healthcare system. In several instances, the prime minister and the health minister were from different political parties. But surprisingly, there was no ideological difference between political parties regarding the NHIP. Thus, the parliament unanimously endorsed the HIA, that allowed the Ministry of Finance (MoF) to release the fund for NHIP [19].

In the federal election of 2017, major political parties (Nepali Congress and Communist Party of Nepal [CPN]) committed (through the election manifesto) to expanding NHIP [65, 66]. In the local election of 2022, two major political parties (Nepali Congress and CPN-Unified Marxist–Leninist [UML]) committed to paying the premium amount for the targeted population [90, 91]. Although there were no ideological differences between the political parties regarding NHIP, there is unhealthy competition between them for claiming credit. Such assertions were evident in the discussion on the Health Insurance Bill at the Federal Parliament, where most of the parliamentary members claimed that the bills would not have been endorsed without their contribution [92].

Although the divergent interests or manipulations towards the NHIP are not vividly observed, it is still doubtful whether the political leadership have clearly understood the concept and relevance, and the subtle difference between BHS and NHIP. For instance, in the budget speech of 2018/2019, the finance minister stated that their government had aimed to expand NHIP to ensure universal coverage of BHS [93], which was a fundamentally incorrect understanding.

The CPN government in 2017 had committed to establishing a 25-bed hospital in each municipality [65]. However, only 14 HFs (nine hospitals and five PHCCs) were established during their tenure of 3.5 years [94]. The same government laid the foundation stone for 309 hospitals. Surprisingly, these public ceremonies were undertaken without adequate preparatory work such as identification of strategic locations, land acquisition or land pooling, or allocation of adequate financial resources. This suggests that the foundation stone ceremony is a political stunt for claiming credit in the future rather than for reforming the health system [95].

### Beneficiaries

**Fig. 7:**
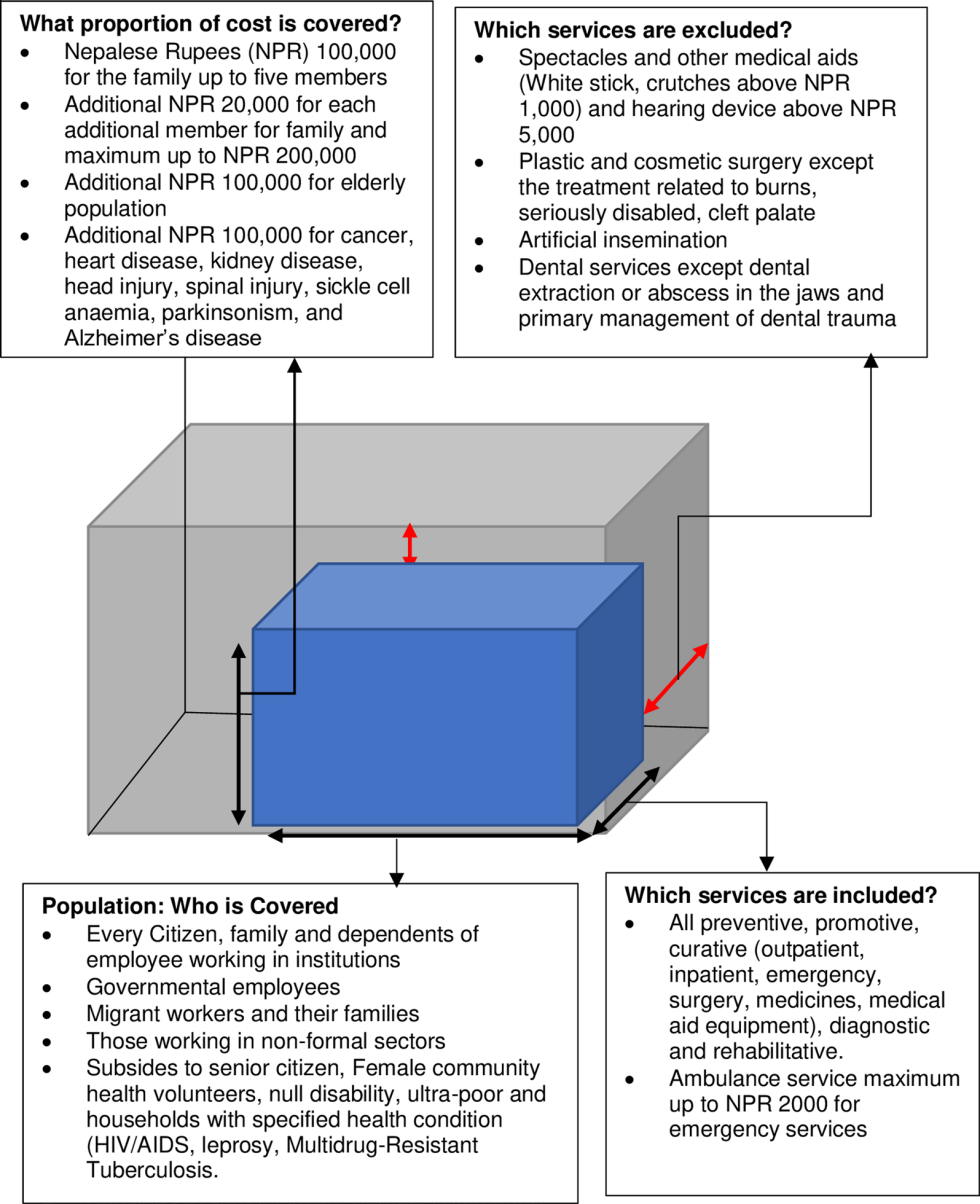
Features of the health insurance programme in Nepal. Source: Prepared by the authors (GNK and RBK), adapted and modified using information from [6, 22, 51]

Figure 7 presents the dimensions of NHIP (population, services and financial protection). Family is the unit of enrolment in NHIP. The minimum premium for a family (of up to five members) is NPR 3500, and an additional NPR 700 for each additional member. The benefit package is up to NPR 100,000 for a family and NPR 20,000 for each additional member. The package includes 1133 types of medicines [24].

As of March 2022, the health insurance policy was active among 74.5% of the total insured population, while more than 1.35 million (25.5% of the insured population) dropped out of the NHIP [25, 69, 70]. The targeted population constituted about 1.33 million insured, which was about one third (33.65%) of the active insured population. In addition, about 2.61 million people enrolled in NHIP by paying the premium (less than 50% of the total insured population) [25]. These figures indicate that few people have enrolled in NHIP through self-payment.

**Fig. 8:**
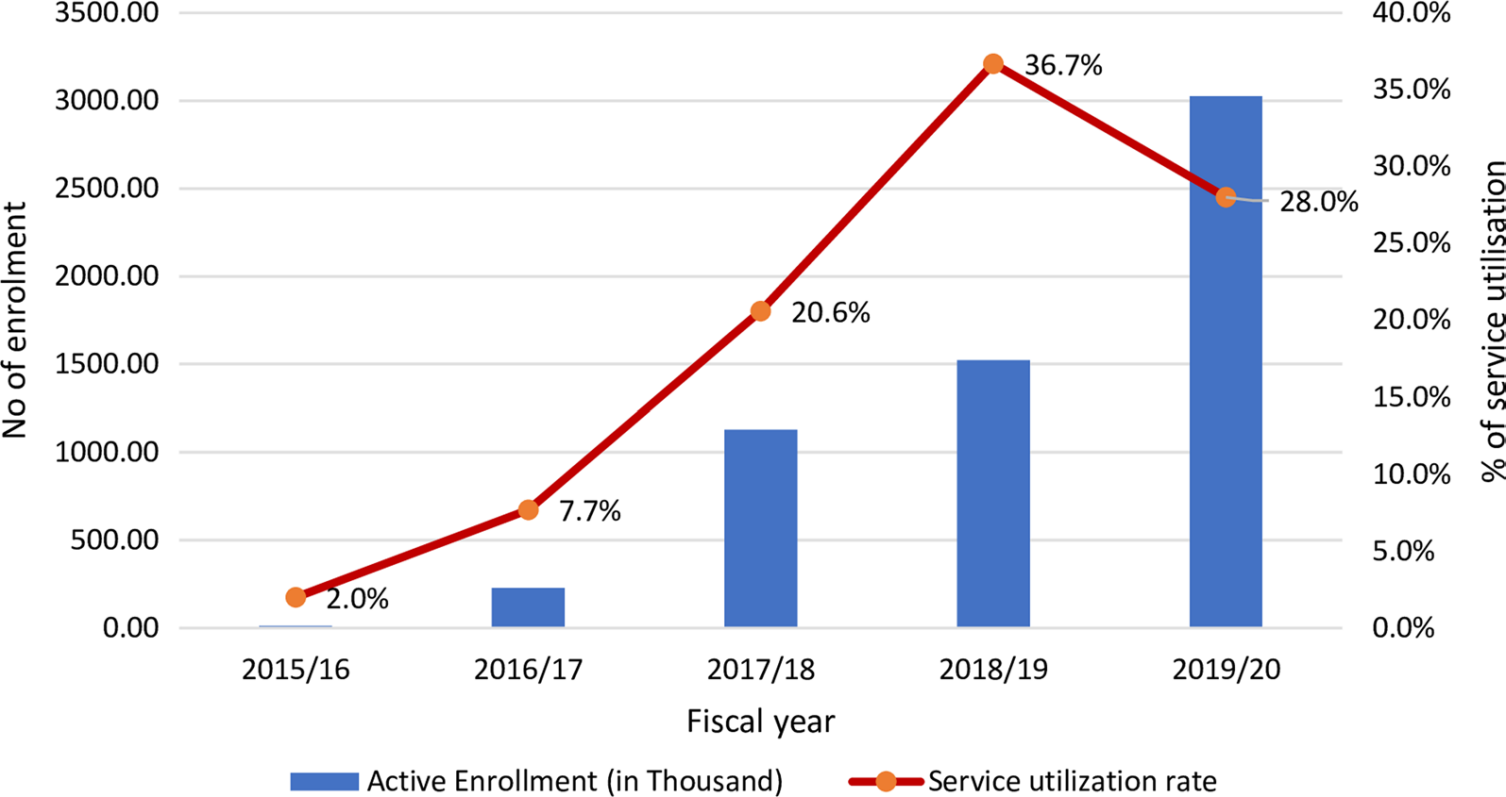
Active enrolment (numbers) versus rate of health services utilization among insured populations in NHIP in Nepal. Source: Prepared by the authors (GNK and RBK), adapted from [51]

Figure 8 shows active enrolment and service utilization rates. It shows that more than a quarter of the insured population have utilized NHIP services in recent years.

Evidence shows that more than 95% of the targeted populations renewed in time if the government paid their premium [48]. In contrast, people tended to drop out of services if they had to pay the premium themselves [48]. Reasons for high drop-out included failure to meet the needs of health insurance services, poor/unsatisfactory experience of care in the past and inadequate benefit packages [25, 71]. The HIA envisioned that the formal sector, corporations (companies, private firms, or cooperatives or similar institutions) and migrant workers and their families would enrol in NHIP [6]. However, there was a lack of clear procedural guidelines, attractive benefits packages, accessible service sites, efficiency in service provision and satisfactory care experience. A media report revealed sick populations, and those needing long-term medical or healthcare services were more interested in enrolling/renewing the insurance policy [48].

### External actors

EDPs have leveraged technical expertise and financial resources in the design, implementation, research, evaluation or policy formulation of the NHIP. For example, the government of the Republic of Korea has supported capacity-building of government officials through Official Development Assistance (ODA) [78]. The German government-supported Agency for International Cooperation (GIZ) has provided technical assistance in IMIS [24]. Earlier, both GIZ and the Korea International Cooperation Agency (KOICA) supported the early rollout and design of the package. They supported the claim management system by providing technical experts including pharmacists, microbiologists and medical officers. GIZ also conducted training on the SOP of the enrolment process, IMIS and health financing. Save the Children supported district-level programme expansion [24]. The International Labour Organization (ILO) supported the actuarial study and capacity-building of HIB staff. However, an extension of technical assistance from EDPs seems volatile after 2021 [96].

Different programmatic ideas have influenced the design of NHIP. For example, GIZ and KOICA primarily advocated a social health insurance model (individual contribution to health through premiums). Their influence was visible in the endorsement of HIA and HIR and the implementation of NHIP during the initial phase. The NHIP provides service beyond the BHS package at an affordable cost through social health protection which is influenced by the German or Korean health insurance model [97]. In contrast, the United Kingdom-funded Nepal Health Sector Programme (NHSP) aims to support the Nepal health sector strategy, including expanding BHS. BHS is a tax-funded programme that resembles the United Kingdom’s National Health Service (NHS) [98]. Anecdotal evidence suggests the government leadership is unclear on the design of these two approaches in the context of heavy reliance on external funding for programmes. External support is crucial to implement NHIP in the context where the informal sector's contribution (62.2% of the total) to the national economy in Nepal [99].

## Discussion

In this study, we sought to document the political economy consideration surrounding NHIP. Findings demonstrate how important political engagement and stakeholder interests are to the formulation and execution of public policy. The NHIP was implemented to protect citizens' right to receive quality healthcare, provide financial protection and improve healthcare-seeking behaviour [6]. However, NHIP has faced organizational and systemic challenges at the policy and implementation levels associated with different political economy factors and interests of stakeholders. Specifically, challenges influencing NHIP included (1) interest groups (e.g. inadequate or delayed reimbursement, hospitals pulling out of NHIP); (2) bureaucracy (hegemony of MoHP over HIB, inadequate staff, and delay of approval of organogram of HIB); (3) budgetary (reimbursement amount has outweighed the collected premium); (4) leadership (political commitments but weak translation into actions to reform the health system, considering poor return on investment [ROI], leaders’ intention for privatization of social health insurance); (5) beneficiaries (compromised quality of healthcare or lack of services when needed, resulting in a high drop-out rate, low interest in renewing the policy by paying the premium themselves); and (6) external actors (technical support in policy design and limited technical support in implementation).

### Interest groups: multiple groups and interests

Several interest groups have been involved in the NHIP. For instance, manual claim review process and delays in claims and reimbursements have possible risks associated with fraudulent claims. About 20 staff are working in the claim review section of HIB where incoming review claims are at least 25,000–30,000 daily [47, 48]. To address the delay in claims and reimbursement, HIB has requested that service providers submit claims within 7 days of treatment [47]. However, this has not been followed strictly because of the opposition from service providers regarding such deadlines [42]. Sometimes the service providers intentionally procrastinate in making claims, and such claims accumulate. In addition, the claim officials cannot verify the claims in detail due to the manual process [47]. As a result, the HIB is unable to detect possible fraudulent claims. Out of NPR 5.11 billion in claims made between September 2020 and June 2021, NPR 282.2 million (5.5% of the total claim amount) was fraudulent [24].

Second, there are policy loopholes in the HIA which indirectly force HIB to purchase suboptimal quality healthcare. For instance, MoHP has the authority to nominate HIB board members. Such members are unable to make any decisions against the interests of MoHP. According to the HIB accreditation guidelines, a minimum service standard (MSS) score of 60% is required for a hospital to be accredited with HIB [37]. However, the dominance of MoHP has hindered HIB in compliance with accreditation guidelines. A government report suggests that less than one third (30%) of public hospitals met minimum requirements [80].

Third, NHIP has been a significant opportunity for private hospitals. The private hospitals act as referral facilities, consequently increasing their revenue (by increasing bed occupancy). In addition, the bed occupancy rate is considered an indicator of seat allocation for medical education in medical colleges [100].

Fourth, the analysis of the top 10 service beneficiaries of NHIP revealed the following (from highest to lowest): essential hypertension, unknown cause of morbidity, abdominal and pelvic pain, non-insulin-dependent diabetes mellitus, disorders of refraction and accommodation, chronic obstructive pulmonary disease, gastritis and duodenitis, and dorsalgia and dental caries [25]. This indicates adverse selection, where people with chronic illness are more attracted to NHIP services [19]. Furthermore, insurees utilize the services (without illness) just because they have health insurance, which indicates the possibility of moral hazard [51]. Such characterization of a moral hazard might be due to the lack of a co-payment provision. Additionally, the provision of cost-sharing (15% in medicines and 25% in breaching referral criteria) in NHIP was removed within 2 years due to beneficiaries’ demand and to reduce the administrative workload of HIB [16]. Our findings were consistent with the studies Ghana [101], Ethiopia [102] and Zambia [103], which reported an increase in healthcare utilization after the implementation of health insurance.

The cost-sharing mechanism in health insurance is a highly debated political agenda [104]. Nepal is not an exception to politicization of cost-sharing in healthcare. The scrapping of co-payment provisions might have resulted in a moral hazard. Co-insurance or co-payment can avoid such behaviour of moral hazard by controlling unnecessary use of services [105].

### Bureaucracy: politics between provider and the purchaser

Recruitment, retention and management of human resources are vital for effective functioning of HIB. First, the HIB lacks a separate human resource structure and is compelled to implement NHIP by limited staff with inadequate implementation capacity and skills. Frequent turnover of temporary staff and EAs was found. Currently, HIB has been operating through temporary secondment staff from MoHP. Consequently, several associated implementation challenges include temporary recruitment of staff under HIB, and termination of such staff anytime [19]. Furthermore, a high drop-out rate of EAs and their passiveness are other enrolment issues [106].

Second, the inspection of quality healthcare is vital for an increase in service coverage. Limited staff influenced the claim reimbursement process with an increased workload in daily administration. Service providers often complain that the HIB reimburses with undue delay or disallowance [38]. Thus, the claim reimbursement section has been unable to perform its activities (e.g. reviewing the benefits package, service costing and timely disbursement) [19]. Consequently, HIB has been unable to inspect and regulate healthcare quality of service providers as provisioned in the HIA [19].

Third, the role of MoHP is hegemonic over HIB, including reliance on daily administrative stuff and coordination with other ministries [6]. For example, HIB requires MoHP’s approval for a permanent organizational structure, although HIB is a legally autonomous body. Recently, HIB submitted the O&M proposal to MoHP for approval, which has not been approved, with no reason given [36]. Such intentional delays impede the effective implementation of NHIP. This “hegemony of providers” over the purchaser has resulted in a fragile relationship between these two institutions [85]. Therefore, experts suspect that a compulsory working arrangement of HIB under MoHP could be detrimental to effective implementation [50].

Major bottlenecks associated with poor expenditure of the allocated budget include a long and bureaucratic procurement processes, inadequate procedural documents (laws and guidelines) and intentional disinterest of staff members. The unspent budget is reclassed in another heading with the vested interests of budgetary authorities [26]. Such budget virement is an example of poor accountability and financial discipline [49]. Thus, the politics of allocating sufficient resources to health insurance has helped leaders politicize the agenda and later allocate the resources based on their interests. The government allocates budgets annually for impoverished households to provide 100% subsidies in premium payment, but it has not yet endorsed any legal documents to identify poor households [25]. Without records of poor households or the procedural documents for identification, political leadership and the bureaucratic system have yet to prepare to provide benefits and entitlements to most marginalized populations [25]. Although HIA and HIR have envisioned different committees at three levels of government, the lack of procedural documents makes them unfunctional [51]. Additionally, SOP to enrol the formal sector has not yet been endorsed [107].

### Budgets: fragmentation, duplication, poor absorption of budgets

Nepal has fragmented health financing schemes and management structures. The vertical publicly funded programmes (e.g. BHS, free healthcare package, safe motherhood programme and impoverished citizens’ service for eight chronic diseases) are managed through federal health budgets [9]. Additionally, there are different health insurance schemes, including NHIP. Before NHIP, there were other, small-scale health insurance programmes (community health insurance, private insurance [voluntary] and insurance for employees of private enterprises) covering a small population. The SSF and EPF also provide health insurance services which cover selected populations working in the formal sector [9].

There are duplications among the packages offered by different health insurance schemes. For instance, SSF, endorsed in 2019, includes sickness schemes covering all inpatient and outpatient treatment in government hospitals [108]. Similarly, the EPF scheme (endorsed in 2020) provides its contributors with a maximum benefits package of up to NPR 100,000 in a fiscal year [39]. Both these benefit packages were endorsed after the implementation of NHIP, and resemble that of NHIP. Such fragmentation of resources and duplication of schemes can reduce the effectiveness of risk redistribution [9]. All these fragmented schemes need to be integrated into a single pool; however, some resistance might arise. Employees and contributors insured through SSF or EPF might show reluctance to enrol in NHIP. The mandatory provision to obtain referral slips from FSPs might trigger such unwillingness.

NHIP reimburses NPR 200 for each outpatient department (OPD) visit in PHCCs, while OPD service in PHCCs is free for those who are not enrolled [80, 109]. Public HFs should provide free BHS; however, HIB has reimbursed more than NPR 2.4 million for comprehensive abortion and post-abortion care services under BHS [61, 110]. This indicates that fragmentation in healthcare financing, duplication of resources and the variation in reimbursement amount between vertical schemes and the NHIP. Such fragmented financing cannot create the larger pool of funds to address the financial hardship. Thus, a single health insurance fund (HIF) to pool different social protection schemes, vertical programmes and public funding of healthcare programmes is needed to reduce healthcare disparity and provide optimal quality healthcare for all.

The reimbursement amount outpacing the collected premium amounts in previous years has signalled a red alarm for financial sustainability [63, 87]. Adverse selection of sick and older people during enrolment and the disinterest of the apparently healthy population in enrolling or renewing the NHIP policy might have created a mismatch. Insurers want to utilize services because they have insurance and demand (sometimes unwanted) prescriptions, referrals and medical tests from FSPs. Some FSPs often refer the patients based on patients’ demand (FSPs receive at least NPR 200 per patient as reimbursement for each OPD visit) rather than providing services which may have led to additional financial burdens [111]. Therefore, special interventions to attract healthy populations and an increase in the renewal of memberships are equally important.

HIB still lacks procedural documents to mobilize the HIF. Thus, more than NPR 6.57 billion that was collected through premium contributions has remained dormant in the bank account [63]. Consequently, HIB must rely on federal sources for its daily operation. Because of this dependence, officials in the MoF are dubious about NHIP. Recently, in an annual budget speech, the government announced the privatization of NHIP. The political leadership might have been interested in privatizing the NHIP after the experience of huge financial liability to reimburse the claims compared to poor ROI. However, the announcement was rescinded after public criticism that privatization could limit the affordability of healthcare among underserved communities who cannot access private and expensive hospitals [112].

### Leadership: high political commitments but missing accountability

NHIP has become a political agenda, with support and commitments from all the major political parties. The Constitution of Nepal (2015) envisioned three tiers of government to formulate necessary legislation, policies or regulations [17]. But strengthening the NHIP from provincial and local level governments is still insufficient. The political leadership have often demonstrated short-sightedness, poor accountability and unhealthy practice to gain political credit.

The previous experience and associated political benefits of the cash-transfer programme might have influenced unhealthy credit claims in NHIP. For instance, the CPN-UML-led government in 1994 provided NPR 100 for the elderly population, which became widely popular and is still helpful for cash votes even after three decades [113]. The governmental announcement (laying the foundation stones) to establish 309 hospitals without ensuring adequate financial resources indicates that political leadership is not adequately prepared to translate their political agendas into actions. Rather, they tend to make popular decisions (without logic and adequate preparation) to gain political benefits [114].

One such irrational announcement in NHIP was seen in setting the enrolment targets. The government had aimed to enrol 100% of the population in NHIP by 2022; however, only 21.4% had enrolled by mid-2022 [25, 26]. Those targets were ambitious and were set without adequate inputs in infrastructure, equipment and human resources [65]. The target was later revised to 40% of the population by 2021 [85] and 50% by 2022 [95]. The revised targets are more realistic than the previous ones; however, increasing the enrolment from 21.4% to 50% in less than a year is still highly ambitious [26].

### Beneficiary: obligation of receiving suboptimal quality health services

Poor availability of services, medicine, equipment or human resource resulted in poor uptake of quality healthcare services among insurees [42]. Reasons for poor quality might be associated with inadequate facility readiness and service availability to implement the NHIP. Many HFs accredited for NHIP face challenges such as the unavailability and readiness of health workers, medicine, infrastructure and equipment [10, 41, 94]. Compromised healthcare quality ultimately led to high drop-out from NHIP [71]. A recent study conducted by HIB reported that more than 40% of complaints were related to service quality and medicines [42]. The insuree had to purchase (full or partial) prescribed medicine or diagnostic services at high costs, although it is included in the benefits package of NHIP [42]. Other grievances included long waiting times, hectic administrative processes [60] and perceived ill behaviour from service providers [42]. On the other hand, people were willing to enrol or renew the services if the quality was improved [20, 71–74, 115].

Inadequate support from doctors and hospitals directly affects the quality of NHIP services. Doctors usually prescribe brand names, which are sometimes unavailable in hospital pharmacies. They prescribe such brand names to obtain financial incentives from pharmaceutical companies. Thus, the insuree are forced to purchase the medicine from private pharmacies.

Many FSPs (usually PHCCs) often act as institutions to issue referral slips [42]. Some FSPs refer patients without providing basic treatment from their level [116], but are unable to fulfil the referral process (issuing referral forms in a given format) [22]. The referral hospitals demand (specific) referral slips issued by the FSPs during each visit. Patients who need multiple visits face several issues due to such requirements [42]. Consequently, many insuree are deprived of referral services and deny renewing their policy. The decision to enrol or renew the policy is related to clients’ satisfaction and their perception that their expectations are fulfilled [71, 75–77, 117]. Therefore, HIB should focus more on the insuree than the service providers [118]. This includes improving the quality of healthcare by revamping the healthcare system.

### External actors: priority and confusion on the model of health financing

Broadly, two categories of EDPs support Nepal’s healthcare financing. First, most EDPs (United States Agency for International Development, United Kingdom’s UK Aid programme, and multilateral agencies) support strengthening the healthcare system by focusing on tax-funded BHS programmes [9]. Funding for BHS from EDPs is channelled through earmarked and sometimes off-budgetary sources. Second, some EDPs (e.g., GIZ, KOICA) support NHIP which is underpinned by the principle of social protection. Under NHIP, revenue sources are member contributions and tax funds (e.g. annual block grants directed to the HIF to subsidize premiums for targeted populations and administrative expenses). Fundamentally, with the tension between the funding support of two different healthcare financial models, there has been confusion among policy-makers. A few EDPs support the NHIP and have minimal technical and financial assistance. The long-term sustainability of NHIP requires leveraging donor agencies' financial and technical expertise [119]. A sector-wide approach has envisioned channelling funds into a common basket which can strengthen the national health system, including NHIP [120].

### Significance of policy and programmes

There are several policy and programmatic recommendations for improving the NHIP. The federal government must introduce separate laws and policies or amend the existing legal provisions to effectively implement NHIP. Firstly, the provision to select FSPs and the mandatory provision to visit FSPs needs to be removed through legal amendments. The mandatory provision for visiting FSPs for general check-up has limited access to NHIP services. Removing such provision can increase access to seeking NHIP services. Secondly, endorsement of different SOPs and guidelines is essential to enrol people in NHIP. Likewise, the benefits package needs to be increased to attract a greater proportion of the population. The renewal rate can be increased through the provision of cost subsides (e.g. gradual top-up/increase in benefit package or subsides in the next annual premium) for those who did not utilize NHIP services throughout the year. Thirdly, implementing a standard medical treatment protocol (SMTP) can be a strategy to control over-prescription. HIB can vet the claims against the SMTP to detect irrational treatment [50]. Fourthly, HIB can use information technology systems (online biometric enrolment, biometric verification at service points, automating electronic claims) to detect fraudulent claims. Integrating electronic medical records and SMTPs in the IMIS platform can enable efficient claim management system [50]. These actions can help to enhance transparency and accountability [121], strengthen robust monitoring systems and reduce administrative workloads [122]. The fifth potential strategy is reintroducing the co-payment system. However, such provision can be exempted for poor and targeted populations. The reintroduction of co-payment can control unnecessary demand/utilization of NHIP services [36].

Sixth, strategic purchasing can offer another approach to strengthen the provider–purchaser split, which can also balance autonomy and accountability. This requires the integration of fragmented health financing schemes into a single pool. HIB needs adequate purchasing power to ensure efficient healthcare service delivery [123]. However, only strong political will, administrative readiness and a highly motivated bureaucratic system can ensure an efficient institutional mechanism. A legal amendment to establish an independent third-party authority (TPA) might be essential to strengthen the provider–purchaser split, which can manage the claim reimbursement process [4]. The TPA can link purchasers and service providers in the claim review process [124]. Consequently, HIB can focus on regulating the quality of healthcare provided by service providers. The effectiveness of TPA in managing health insurance has been reported in other countries like India [125] and Kenya [126].

**Fig. 9:**
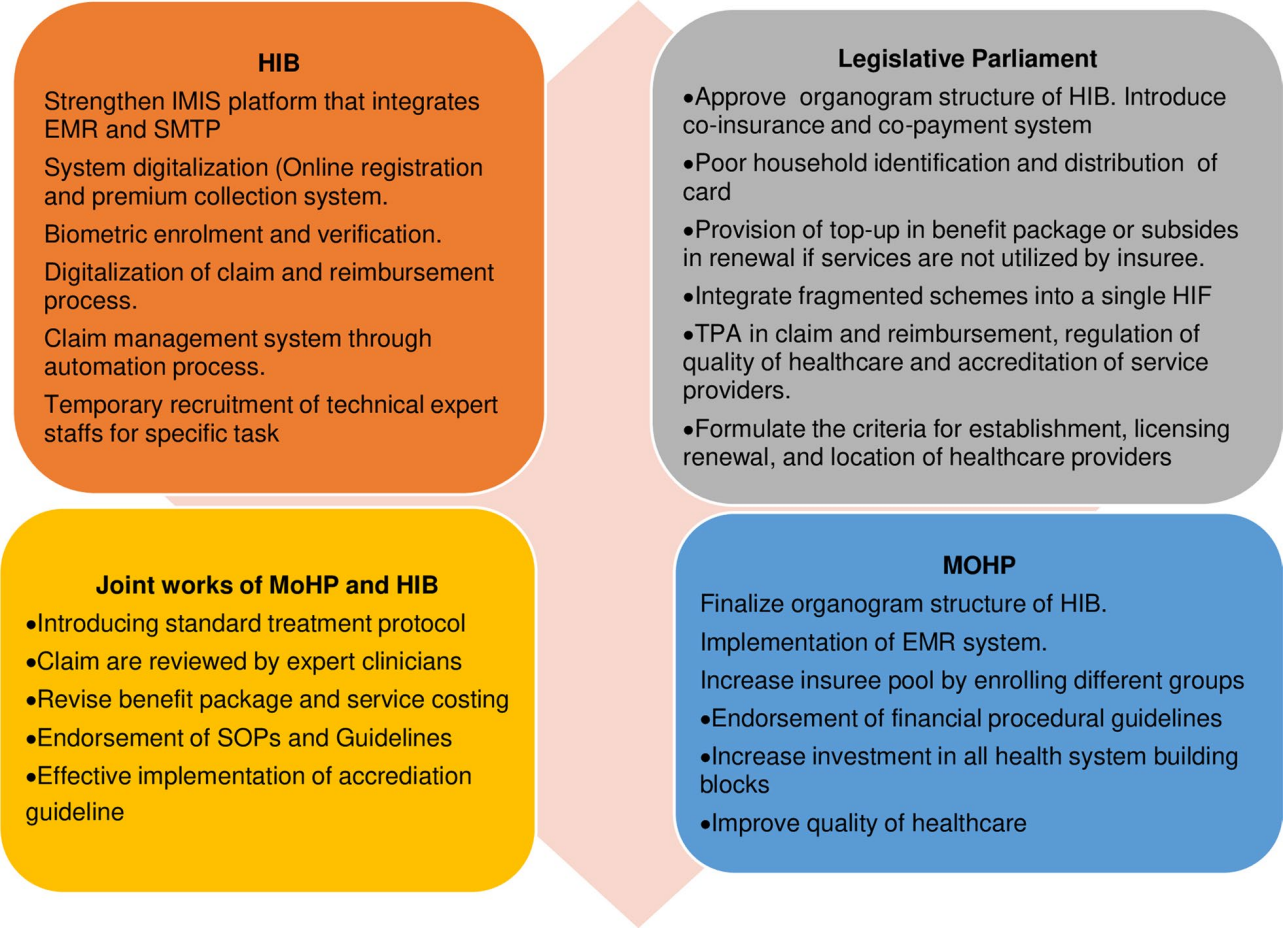
Possible strategies for strengthening NHIP and programme performance

Finally, HIB has envisioned a supportive role from the provincial and local governments. But their ownership is still inadequate. The health insurance coordination committees at the provincial and local levels are not sufficiently functional to accomplish their expected roles as envisioned in the HIA [25]. Some provincial and local governments have started to enrol people in the NHIP; however, there are numerous opportunities to do more on policy and insurance programme implementation [19, 25]. Local governments can support in the identification of poor households, which are estimated to be almost a quarter of the total population [127]. The media and civil society had heavily criticized the process adopted in identifying the poor households after influential community people captured the subsidies in the name of the poor [45]. Local governments can use a participatory rural appraisal approach to identify poor families, which was practised in Indonesia [128], Ghana [129], India [130] and Latin America [131]. Local governments can contribute premium amounts for extremely poor people [25]. They can also conduct demand-generating activities (raising awareness, strengthening trust, motivating enrolment and renewal), procurement (essential medicine, equipment and logistics), accountability regulatory mechanisms, and tracking of progress [132]. However, anecdotal evidence suggests that local governments are sometimes reluctant to conduct demand-generating activities at the community level. They anticipate a possible threat of “boomerang” after mass enrolment in the NHIP if they cannot provide quality healthcare. The inability to provide quality healthcare even after prepayment of premiums can backfire on local government leadership [19]. Establishing local hospitals (at least one) in each municipality is vital to ensure the availability of NHIP services at the local level. The local government can recruit medical officers, establish pharmacy and lab facilities to obtain accreditation for the NHIP. Figure 9 presents possible strategies for authorities and stakeholders to strengthen the NHIP in Nepal.

### Strengths and limitations

This is the first review of the NHIP of Nepal conducted using secondary data from several sources and explained using a political economy of health as a guiding framework. Findings were also validated by experts working in the arena of concern. From a methodological perspective, this study can provide insights into how literature review and expert opinion can be integrated to obtain a big picture of the problem. This study has some limitations. We could not include the direct voice of beneficiaries and stakeholders of NHIP. However, this study provided a comprehensive overview of Nepal's health insurance programme. Primary studies can address specific interest groups or stakeholders of Nepal’s health insurance programme. This qualitative synthesis of available evidence included a wide range of studies, reports and mostly grey literature on NHIP. Having some evidence is better than having no evidence. Therefore, the findings of this review provide insights for future research on the policy and implementation of health insurance programmes in Nepal and countries with similar socioeconomic status and health systems.

## Conclusions

Despite enabling policy environment for the health insurance programme, the NHIP has not shown promising results. Several organizational and systematic challenges have hindered the policy implementation and service coverage, linked with many interest groups/stakeholders. Programme performance can be strengthened using multiple reform strategies. Firstly, bureaucratic reforms like digitalization of claims and reimbursement (development of apps), endorsement of the organizational structure of HIB, recruitment of HIB staff, development of a robust information management system and digital technology could help maximize the information system efficiency. Secondly, reforming service delivery by increasing benefit packages and tripartite agreements for accreditation of HFs to ensure quality healthcare delivery would be helpful. Additionally, enrolment under NHIP needs to be made universal, whether working in formal or informal sectors. The provision of compulsory enrolment in the programme and health services beyond BHS packages could balance the demand and supply of services, including trust-building between providers and patients. Thirdly, revision of the financial model is vital, including introduction of a cost-sharing/co-payment model. Cost standardization and increased service delivery platforms can improve the supply of health services, resulting in easy access. The government should consolidate the fragmented financial schemes and mainstream them to make a single payment system to avoid duplication.

Furthermore, a robust financial monitoring system can be introduced to monitor the budget flow. Finally, leveraging technical expertise and financial resources on NHIP would aid in uniformity in the modality of NHIP operation, maximizing its benefits. NHIP was an important breakthrough in reducing OOPs and improving access to quality health services to realize UHC. Achieving improved NHIP programme performance and improved access to insurance programmes requires high public commitment and accountability from the political and bureaucratic spheres.

## Disclaimer

The findings presented in this article are based solely on the available literature. Interpretation and reflection presented in the paper are of the authors and do not represent the views, interests or funded work of the organizations where the authors are affiliated.

## Data Availability

Data used in this study are included in this manuscript.

## Abbreviations

BHS: Basic healthcare services
BPKIHS: B.P. Koirala Institute of Health Sciences
CHE: Current health expenditure
CPN: Communist Party of Nepal
CPN-UML: Communist Party of Nepal (Unified Marxist–Leninist)
EAs: Enrolment assistants
EDPs: External development partners
EPF: Employees Provident Fund
FSPs: First service points
GIZ: German Agency for International Cooperation
HFs: Health facilities
HIA: Health Insurance Act
HIB: Health Insurance Board
HIF: Health Insurance Fund
HIR: Health Insurance Regulation
HP: Health post
ILO: International Labour Organization
IMIS: Insurance management information system
KOICA: Korea International Cooperation Agency
MoF: Ministry of Finance
MoHP: Ministry of Health and Population
NHIP: National Health Insurance Program
NHSP: Nepal Health Sector Programme
NPR: Nepalese rupees
O&M: Organization and management
ODA: Official development assistance
OOP: Out-of-pocket
OPD: Outpatient department
PHCCs: Primary healthcare centres
SDIP: Safe Delivery Incentive Program
SHSDC: Social Health Security Development Committee
SMTP: Standard medical treatment protocol
SOP: Standard operating procedure
SSF: Social Security Fund
TPA: Third-party authority
UHC: Universal health coverage

## References

[1] Sharma PR (2005). Nepal’s quest for health. J Inst Med Nepal.

[2] Mahat A, Citrin D, Bista H. NGOs, partnerships, and public-private discontent in Nepal’s health care sector. Med Anthropol Theory. 2018; 5(2).

[3] Magar A. National health policy of Nepal-time to revisit and reform. J Nepal Med Assoc. 2013; 52(190). 24362675

[4] Sharma J, Aryal A, Thapa GK (2018). Envisioning a high-quality health system in Nepal: if not now, when?. Lancet Glob Health.

[5] Ministry of Finance. Economic Survey (Unofficial Translation) In*.* Kathmandu, Nepal 2021. https://www.mof.gov.np/uploads/document/file/1633341980_Economic%20Survey%20(Engslish)%202020-21.pdf.

[6] Government of Nepal. Health Insurance Act (Unofficial Translation) In*.*; 2017. https://hib.gov.np/public/uploads/shares/notice_hib/health-insurance-act-2074.pdf.

[7] Government of Nepal. Intergovernmental Fiscal Arrangement Act, 2017 In*.*; 2017. https://mof.gov.np/uploads/document/file/An%20Act%20Made%20to%20Intergovernmental%20Fiscal%20Arrangement%20final%2011-15_20180305120026.pdf.

[8] Ministry of Health and Population and Nepal Health Sector Support Programme. Budget Analysis of Ministry of Health FY 2017/18 In: *Ministry of Health and Population, Nepal Health Sector Support Programme.* Kathmandu 2018.

[9] Ministry of Health and Population. Situation analysis of health financing in Nepal. In*.* Kathmandu, Nepal. MoHP, World Bank, WHO, GIZ, 2019.

[10] Ministry of Health Nepal, New ERA Nepal, Nepal Health Sector Support Program (NHSSP), ICF: Nepal Health Facility Survey 2015. In*.* Kathmandu. Nepal: Ministry of Health Nepal 2015. Retrieved from https://dhsprogram.com/pubs/pdf/SPA24/SPA24.pdf.

[11] Gurung G, Gauld. Private gain, public pain: does a booming private healthcare industry in Nepal benefit its people? In., vol. 2021; 2016. https://blogs.bmj.com/bmj/2016/09/30/does-a-booming-private-healthcare-industry-in-nepal-benefit-its-people/.

[12] Ministry of Health and Population. Nepal National Health Accounts 2017/18. In*.*; 2022. Retrieved from http://apps.who.int/nha/country/npl/nepalnha.pdf?ua=1.

[13] Khanal GN (2019). Conditional cash transfer policies in maternal health service utilization in Nepal: analysis of safe delivery incentive program (Aama Surakshya Karyakram) using Kingdon’s multiple streams framework. Int J Health Plann Manage.

[14] Ranabhat CL, Kim CB, Singh A, Acharya D, Pathak K, Sharma B, Mishra SR (2019). Challenges and opportunities towards the road of universal health coverage (UHC) in Nepal: a systematic review. Arch Public Health.

[15] Witter S, Khadka S, Nath H, Tiwari S (2011). The national free delivery policy in Nepal: early evidence of its effects on health facilities. Health Policy Plan.

[16] Social Health Security Development Committee. Social Health Security Programme Operating Rules, 2014. Kathmandu, Nepal. In.; 2014.

[17] Government of Nepal. Constitution of Nepal 2015. In: *Kathmandu: Secretariat of Constituent Assembly, Singha Durbar.* 2015.

[18] Government of Nepal. National Health Insurance Policy (Unofficial Translation). In*.* Kathmandu, Nepal; 2013. https://hib.gov.np/public/uploads/shares/health_insurance_2071_policy.pdf.

[19] Gurung GB, Panza A (2022). Implementation bottlenecks of the National Health Insurance program in Nepal: Paving the path towards Universal Health Coverage: a qualitative study. Int J Health Plann Manage.

[20] Ghimire P, Sapkota VP, Poudyal AK (2019). Factors Associated with Enrolment of Households in Nepal’s National Health Insurance Program. Int J Health Policy Manag.

[21] Government of Nepal. Health Insurance Board Dashboard. Retrieved June 28, 2021, from https://dashboard.hib.gov.np/. In*.*; 2020.

[22] Government of Nepal. Health Insurance Regulation (Unofficial Translation) In*.*; 2019. https://hib.gov.np/public/uploads/shares/notice_hib/health-insurance-regulation-2075.pdf.

[23] Health Insurance Board. Health Insurance Training Manual In*.* Kathmandu, Nepal: Health Insurance Board 2017.

[24] Health Insurance Board. Annual report of Health Insurance Board 2020/2021. In*.* Kathmandu Nepal Health Insurance Board; 2020/2021. https://hib.gov.np/public/uploads/shares/notice_hib/annual-report-2077-078.pdf.

[25] Gyanwali S. Status and progress of social health insurance scheme in Nepal with challenges and opportunities. In: *8th National Virtual Summit of Health and Population Scientists in Nepal* 2022.

[26] Government of Nepal. Budget Speech 2019/20 (Unofficial Translation). In*.*; 2019.

[27] Adhikari B, Mishra SR, Schwarz R (2022). Transforming Nepal’s primary health care delivery system in global health era: addressing historical and current implementation challenges. Glob Health.

[28] Khatri RB. Towards equity of maternal and newborn health services in Nepal. PhD dissertation, submitted to the University of Queensland, Brisbane, Australia; 2021. 10.14264/c718705

[29] Karkee R, Lee AH, Binns CW (2015). Bypassing birth centres for childbirth: an analysis of data from a community-based prospective cohort study in Nepal. Health Policy Plan.

[30] Mehata S, Paudel YR, Dariang M, Aryal KK, Paudel S, Mehta R, King S, Barnett S (2017). Factors determining satisfaction among facility-based maternity clients in Nepal. BMC Pregnancy Childbirth.

[31] Karkee R, Comfort J (2016). NGOs, foreign aid, and development in Nepal. Front Public Health.

[32] Rizvi SS, Douglas R, Williams OD, Hill PS (2020). The political economy of universal health coverage: a systematic narrative review. Health Policy Plan.

[33] Agarwal S, Kirk K, Sripad P, Bellows B, Abuya T, Warren C (2019). Setting the global research agenda for community health systems: literature and consultative review. Hum Resour Health.

[34] Sparkes SP, Bump JB, Ozcelik EA, Kutzin J, Reich MR (2019). Political economy analysis for health financing reform. Health Syst Reform.

[35] Campos PA, Reich MR (2019). Political analysis for health policy implementation. Health Syst Reform.

[36] Health Insurance Board. Annual Policy and Program 2022/2023 (Unofficial Translation) In*.* Kathmandu, Nepal; 2022. https://hib.gov.np/public/uploads/shares/hib-annual-policy-program-2079-80.pdf.

[37] Health Insurance Board. Accreditation guideline for service providing facilities, 2021 (in Nepali) In*.* Kathmandu, Nepal; 2021. https://hib.gov.np/public/uploads/shares/hospital_listing_guidelines_2078.pdf.

[38] National Planning Commission. Access to free basic and emergency health services as envisioned in the constitution. In: *National Dialogue, Health sector in Nepal: Present Status and future Course of Action.* Kathmandu, Nepal; 2022. https://www.youtube.com/watch?v=TaH-idqb2t0&t=10140s.

[39] Employee Provident Fund. Procedural guidelines for contributors to implement of healthcare plan. In*.* Kathmandu, Nepal, 2021.

[40] Gurung G, Koirala S. Dangers of privatising health insurance in Nepal. In: *Nepali Times* Kathmandu, Nepal: Himalmedia Pvt Ltd | Patan Dhoka; 2022. https://www.nepalitimes.com/opinion/dangers-of-privatising-health-insurance-in-nepal/.

[41] Mishra SR, Khanal P, Karki DK, Kallestrup P, Enemark U (2015). National health insurance policy in Nepal: challenges for implementation. Glob Health Action.

[42] German Agency for International Cooperation. Analysis of Complain and Response Mechanism (CRM) of Health Insurance Board (Unpublished Report). In. Kathmandu, Nepal; German Agency for International Cooperation; 2022.

[43] Sapkota VP, Bhusal UP (2017). Governance and purchasing function under social health insurance in Nepal: looking back and moving forward. J Nepal Health Res Counc.

[44] Health Insurance Board: Press Release (in Nepali). Retrieved from https://hib.gov.np/public/uploads/shares/press-release-hib-2079-03-09.pdf.

[45] Shahi N. Preying on Poor People’s ID. In*.* Kathmandu, Nepal.: Centre of Investigative Journalism; 2018. https://cijnepal.org.np/preying-on-poor-peoples-id/.

[46] Health Insurance Board. Staff List 2022. In*.*, vol. 2022; 2022. https://hib.gov.np/en/pages/staffs.

[47] Health Insurance Board. Circular on Claim and Reimbursement (in Nepali) (Reference Number 789) In*.* Kathmandu, Nepal; 2022. https://hib.gov.np/public/uploads/shares/notice-claim-documents-related-hf.pdf.

[48] Chaulagain P. Poor quality, cumbersome process: Danger of neglect of health insurance program (In Nepali) In: *Onlinekhabar.* Kathmandu, Nepal: Onlinekhabar 2022. https://www.onlinekhabar.com/2022/04/1104207.

[49] National Planning Commission. Role of private sector and civil society in strengthening health service delivery. In: *National Dialogue, Health sector in Nepal: Present Status and future Course of Action.* Kathmandu, Nepal; 2022. https://www.youtube.com/watch?v=TaH-idqb2t0&t=10140s.

[50] Basaula D, Adhikari SR. Plenary Session I Strengthening social health protection in Nepal: the role of evidence towards universal health coverage. In: *Nepal’s Health Sector: Policy, planning and Implementation.* Kathmandu, Nepal; 2022.

[51] Paudel KP. Nepal’s Health Sector: Policy, planning and Implementation. National Dialogue, Health sector in Nepal: Present Status and future Course of Action. In*.* Kathmandu Nepal; 2022. https://www.youtube.com/watch?v=TaH-idqb2t0&t=18797s.

[52] Health Insurance Board. Directive on the amendments of rates of service package In*.* Kathmandu, Nepal 2022. https://hib.gov.np/public/uploads/shares/benfits-package-partial-update-notice.pdf.

[53] Health Insurance Board. Partial benefit package In*.* Kathmandu, Nepal 2021. https://hib.gov.np/public/uploads/shares/partial-benefit-package-update-list.pdf.

[54] Health Insurance Board. List of service providing health facilities under NHIP. In*.* Kathmandu, Nepal Health Insurance Board. 2021. https://hib.gov.np/public/uploads/shares/hf-list-latest-feb202.

[55] Ministry of Health and Population. Nepal National Health Accounts 2009/10–2011/12. In*.*; 2016.

[56] Ministry of Health and Population. Nepal National Health Accounts 2012/13-2015/16. Kathmandu, Nepal. In*.*; 2018.

[57] Pokharel R, Silwal PR (2018). Social health insurance in Nepal: A health system departure toward the universal health coverage. Int J Health Plann Manage.

[58] Government of Nepal. Budget Details: Red Book (Fiscal year 2020/21). Kathmandu. In*.*; 2020.

[59] Government of Nepal. Budget Details: Red Book (Fiscal year 2021/22). Kathmandu. In*.*; 2021.

[60] Ministry of Finance. Forecasting of expenditure and budget heading (Nepali). In*.* Kathmandu, Nepal; 2021. Retrieved from https://www.mof.gov.np/public/uploads/document/file/Redbook%20Press%20(Final)_20210530065617.pdf.

[61] World Health Organization. Health financing mechanism including free schemes for sexual and reproductive health services in Nepal. An assessment of health financing for sexual and reproductive health. In*.* Kathmandu: World Health Organization, Country Office for Nepal; 2020.

[62] Office of the Auditor General. 57th Annual Report of Auditor General (Unofficial Translation). In*.*; 2020. https://oag.gov.np/menu-category/926/en.

[63] Office of the Auditor General. 59th Annual Report of Auditor General (Unofficial Translation). In*.* Kathmandu, Nepal: Office of the Auditor General. 2022.

[64] Office of the Auditor General. 56th Annual Report of Auditor General (Unofficial Translation). Kathmandu, Nepal. In*.*; 2019. https://oag.gov.np/menu-category/926/en.

[65] Communist Party of Nepal (CPN). Menifesto of Communist Party of Nepal: Election of House of Representatives and Provincial Council 2017 (Unofficial translation). In*.*; 2017.

[66] Nepali Congress. Menifesto of Nepali Congress: Election for Members of Parliament and Provincial Council 2017 (Unofficial Translation). In*.*; 2017.

[67] Government of Nepal. Economic Survey 2020/2021 (Unofficial Translation). In*.* Kathmandu, Nepal Ministry of Finance. 2021.

[68] Bhusal UP, Sapkota VP (2021). Predictors of health insurance enrolment and wealth-related inequality in Nepal: evidence from Multiple Indicator Cluster Survey (MICS) 2019. BMJ Open.

[69] Ranabhat CL, Subedi R, Karn S (2020). Status and determinants of enrollment and dropout of health insurance in Nepal: an explorative study. Cost Eff Resour Alloc.

[70] Sharma P, Yadav DK, Shrestha N, Ghimire P. Dropout Analysis of a National Social Health Insurance Program at Pokhara Metropolitan City, Kaski, Nepal. Int J Health Policy Manag 2021. 10.34172/ijhpm.2021.171 PMC9818104 35042322

[71] Gurung GB, Panza A (2022). Predictors of annual membership renewal to increase the sustainability of the Nepal National Health Insurance program: a cross-sectional survey. PLOS Global Public Health.

[72] Adhikari M, Gahatraj N. Willingness and status of social health insurance among people of Pokhara Lekhnath, Nepal. 2020.

[73] Joshi SK, Joshi DK, Joshi SK, Adhikari N, Joshi S, Joshi DR. Effect of Health Insurance Program in Social Security in Kailali District, Nepal. Acta Sci Pharm Sci. 2020, 4(3).

[74] Acharya D, Devkota B, Adhikari R (2018). Willingness to pay for family health insurance: evidence from Baglung and Kailali districts of Nepal. Glob J Health Sci.

[75] Shrestha MV, Manandhar N, Dhimal M, Joshi SK (2020). Awareness on social health insurance scheme among locals in Bhaktapur Municipality. Hindu.

[76] Sharma S, Banjara S (2020). Perception of Social Health Insurance Program among Community People in Pokhara, Nepal. Janapriya J Interdiscip Stud.

[77] Acharya D, Devkota B, Gautam K, Bhattarai R (2020). Association of information, education, and communication with enrolment in health insurance: a case of Nepal. Arch Public Health.

[78] Diwas Acharya. Impact of Official Development Assistance on Capacity Development: A Case Study for Nepal. Seoul National University 2017.

[79] Shrestha J. Towards Universal Health Coverage: an analysis of health insurance program of Nepal. In: *NHRC summit presentation* Kathmandu, Nepal; 2022.

[80] Department of Health Services. Annual Report 2076/77 (2019/20). In*.* Kathmandu, Nepal; 2020.

[81] Republica Nepal. BPKIHS desperately waits for health ministry to pay it over Rs 260 million in health insurance. In*.*; 2022. https://myrepublica.nagariknetwork.com/news/bpkihs-desperately-waits-for-health-ministry-to-pay-it-over-rs-260-million-in-health-insurance/.

[82] Mahima Devkota. Hospitals pulling out of health insurance program. In: *The Rising Nepal.* Kathmandu, Nepal; 2022. https://risingnepaldaily.com/news/13041.

[83] Health Insurance Board. Proposed Temporary Structure of Health Insurance Board. In*.* Kathmandu, Nepal: Health Insurance Board. 2022.

[84] Khatri RB, Durham J, Assefa Y (2021). Utilisation of quality antenatal, delivery and postnatal care services in Nepal: an analysis of Service Provision Assessment. Global Health.

[85] Government of Nepal. Budget Speech 2020/21 (Unofficial Translation). In*.*; 2020.

[86] Subedi SR. Multi-billion-rupee health insurance scheme in crisis. In: *myRepublica* 2019. https://myrepublica.nagariknetwork.com/news/multi-billion-rupee-health-insurance-scheme-in-crisis/.

[87] Office of the Auditor General. 58th Annual Report of Auditor General (Unofficial Translation). In*.* Kathmandu, Nepal. 2021. https://oag.gov.np/menu-category/926/en.

[88] Jones S (2012). The politics of social rights. Public Manag Rev.

[89] Thapa PB, Khanal GN. Ensuring our health. Republica Daily. In*.*; 2017. https://myrepublica.nagariknetwork.com/news/ensuring-our-health/.

[90] CPN-UML. Manifesto of Nepal Communist Party (Unified Marxist, Leninist) for local election 2022 (Unofficial translation) In. Kathmandu, Nepal; 2022. https://drive.google.com/file/d/1AUrehH7-xdVS42j4-BlC_hBgluq4R0L4/view.

[91] Nepali Congress. Commitment of Nepali Congress for Local Election 2022 (unofficial translation) In*.* Kathmandu, Nepal; 2022. https://api.nepalicongress.org/uploads/documents/pdf/ncdocuments9132705481822-1651659252145.pdf.

[92] Legislature Parliament of Nepal. Parliamentary Discussion on Health Insurance Bill (2017). In*.* Kathmandu, Nepal; 2017. https://www.youtube.com/@legislatureparliamentnepal5785/search?query=9%20Aug%202017.

[93] Government of Nepal. Budget Speech of Fiscal Year 2018/19 In*.* Kathmandu, Nepal; 2019. https://www.mof.gov.np/uploads/document/file/Budget_Speech_2018_20201118080436.pdf.

[94] Government of Nepal. Economic Survey 2021/2022(Unofficial Translation). In*.* Kathmandu, Nepal Ministry of Finance. 2021. https://www.mof.gov.np/site/publication-category/21.

[95] Ministry of Finance. Public Announcement of Income-Expenditure Details of Fiscal Year 2021/22 (Unofficial Translation). In*.* Kathmandu, Nepal; 2022. https://www.mof.gov.np/uploads/document/file/1627455031_Budget_speech_2021.pdf.

[96] Health Insurance Board. Sharing Meeting with External Development Partners on Health Insurance Program. In Situation analysis of Health Insurance Program. In. Kathmandu.; 2020.

[97] Ministry of Health and Population. Nepal health sector strategy 2016–2021. In. Kathmandu: Ministry of Health, Government of Nepal; 2015.

[98] Charlesworth A, Bloor K (2018). 70 years of NHS funding debate: how do we know how much is enough?. BMJ.

[99] Central Bureau of Statistics. Report on the Nepal Labour Force Survey. In*.* Kathmandu, Nepal; 2017/18.

[100] Medical Education Commission. National Medical Education Regulation. In*.* Kathmandu, Nepal; 2020. http://www.mec.gov.np/public/uploads/shares/ActandRegulation/mec_regulation.pdf.

[101] Dalinjong PA, Laar AS (2012). The national health insurance scheme: perceptions and experiences of health care providers and clients in two districts of Ghana. Heal Econ Rev.

[102] Atnafu DD, Tilahun H, Alemu YM (2018). Community-based health insurance and healthcare service utilisation, North-West, Ethiopia: a comparative, cross-sectional study. BMJ Open.

[103] Masiye F, Kaonga O, Kirigia JM (2016). Does user fee removal policy provide financial protection from catastrophic health care payments? Evidence from Zambia. PLoS ONE.

[104] Kiil A, Houlberg K (2014). How does copayment for health care services affect demand, health and redistribution? A systematic review of the empirical evidence from 1990 to 2011. Eur J Health Econ.

[105] World Health Organization. Universal health coverage: moving towards better health: action framework for the Western Pacific Region. In. Manila: WHO Regional Office for the Western Pacific; 2016

[106] Nepal Health Research Council. Assessment of Social Health Insurance scheme in selected districts of Nepal. In. Kathmandu, Nepal; 2018

[107] Health Insurance Board. Acts, Regulations, Directives, and Procedures List 2022. In., vol. 2022; 2022. https://hib.gov.np/en/category/regulation.

[108] Social Security Fund. Guidelines on selection of health facility and reimbursement process of social security plan (2019) (Unofficial Translation). In*.* Kathmandu, Nepal; 2019. https://ssf.gov.np/list/act_regulation/savasathaya-sasatha-chhanata-tatha-yajanaka-rakama-bhakatana-samabnathha-karayavathha.

[109] Health Insurance Board. Description of services and Rate of benefit package. In*.* Kathmandu, Nepal; 2017. https://hib.gov.np/public/uploads/shares/notice_hib/benefir-package.pdf.

[110] Ministry of Health and Population. Public Health Service Regulation (Unofficial translation). In*.* Kathmandu, Nepal; 2020 https://drive.google.com/file/d/1STq4iCAhqyrsXmnXmLxItn1dvXZtGmRM/view.

[111] Health Insurance Board. Circular on Claiming the amount of referral (in Nepali) (Reference Number 628) In*.* Kathmandu, Nepal; 2022 https://hib.gov.np/public/uploads/shares/notice_hib/hospital-notice-referal-cases.pdf.

[112] Sonia Awale. Ensuring health insurance for all Nepali. In: *Nepali Times.* Kathmandu, Nepal; 2022. https://www.nepalitimes.com/banner/ensuring-health-insurance-for-all-nepalis/.

[113] Drucza K (2017). The politics behind social protection in Nepal. Asian J Comp Polit.

[114] Savedoff WD, de Ferranti D, Smith AL, Fan V (2012). Political and economic aspects of the transition to universal health coverage. Lancet.

[115] Kazaura M, Kamazima SR (2021). Knowledge, attitudes and practices on tuberculosis infection prevention and associated factors among rural and urban adults in northeast Tanzania: a cross-sectional study. PLOS Global Public Health.

[116] Health Insurance Board. Circular on claim amount of referral services (in Nepali) (Reference Number 628) In*.* Kathmandu, Nepal; 2022 https://hib.gov.np/public/uploads/shares/notice_hib/hospital-notice-referal-cases.pdf.

[117] Boateng D, Awunyor-Vitor D (2013). Health insurance in Ghana: evaluation of policy holders’ perceptions and factors influencing policy renewal in the Volta region. Int J Equity Health.

[118] Kruk ME, Gage AD, Arsenault C, Jordan K, Leslie HH, Roder-DeWan S, Adeyi O, Barker P, Daelmans B, Doubova SV (2018). High-quality health systems in the Sustainable Development Goals era: time for a revolution. Lancet Glob Health.

[119] Tangcharoensathien V, Patcharanarumol W, Ir P, Aljunid SM, Mukti AG, Akkhavong K, Banzon E, Huong DB, Thabrany H, Mills A (2011). Health-financing reforms in southeast Asia: challenges in achieving universal coverage. Lancet.

[120] Ministry of Finance. An Assessment of Sector Wide Approach (SWAp) in the Health and Education Sectors of Nepal. In*.* Kathmandu, Nepal; 2018 [Retrieved from https://mof.gov.np/uploads/document/file/SWAP Study_20190426050525.pdf].

[121] Kiri VA, Ojule AC (2020). Electronic medical record systems: a pathway to sustainable public health insurance schemes in sub-Saharan Africa. Niger Postgrad Med J.

[122] Atuoye KN, Vercillo S, Antabe R, Galaa SZ, Luginaah I (2016). Financial sustainability versus access and quality in a challenged health system: an examination of the capitation policy debate in Ghana. Health Policy Plan.

[123] World Health Organization. Strategic purchasing for universal health coverage: key policy issues and questions: a summary from expert and practitioners’ discussions. Health Financing Working Paper no 8. In*.*; 2017

[124] Bhat R, Babu SK. Health insurance and third party administrators: issues and challenges. *Econ Polit Wkly* 2004:3149–3159.

[125] Aggarwal A (2010). Impact evaluation of India's ‘Yeshasvini’ community-based health insurance programme. Health Econ.

[126] Bundi G (2012). Role of third party administrators in setting up managed health care systems: a case of Henner Groupe Kenya.

[127] National Planning Commission. Nepal Multidimensional Poverty Index 2018: Analysis Towards Action. In*.* Kathmandu, Nepal. National Planning Commission; 2018.

[128] Banerjee A, Hanna R, Kyle J, Olken BA, Sumarto S (2018). Tangible information and citizen empowerment: identification cards and food subsidy programs in Indonesia. J Polit Econ.

[129] Aryeetey GC, Jehu-Appiah C, Spaan E, D'Exelle B, Agyepong I, Baltussen R (2010). Identification of poor households for premium exemptions in Ghana’s National Health Insurance Scheme: empirical analysis of three strategies. Trop Med Int Health.

[130] Banerjee A, Duflo E, Chattopadhyay R, Shapiro J. Targeting Efficiency: How well can we identify the poorest of the poor? *Institute for Financial Management and Research Centre for Micro Finance Working Paper* 2009, 21.

[131] Karlan D, Thuysbaert B (2019). Targeting ultra-poor households in Honduras and Peru. The World Bank Economic Review.

[132] Yuan B, Jian W, He L, Wang B, Balabanova D (2017). The role of health system governance in strengthening the rural health insurance system in China. Int J Equity Health.

